# Observation and Modeling of a Sharp Oxygen Threshold in Aqueous Free Radical and RAFT Polymerization

**DOI:** 10.1021/acs.jpcb.2c06067

**Published:** 2022-12-15

**Authors:** Julia S. Siqueira, Matthew Crosley, Wayne F. Reed

**Affiliations:** † Tulane University, New Orleans, Louisiana70118, United States; ‡ Fluence Analytics, Stafford, Texas77477, United States

## Abstract

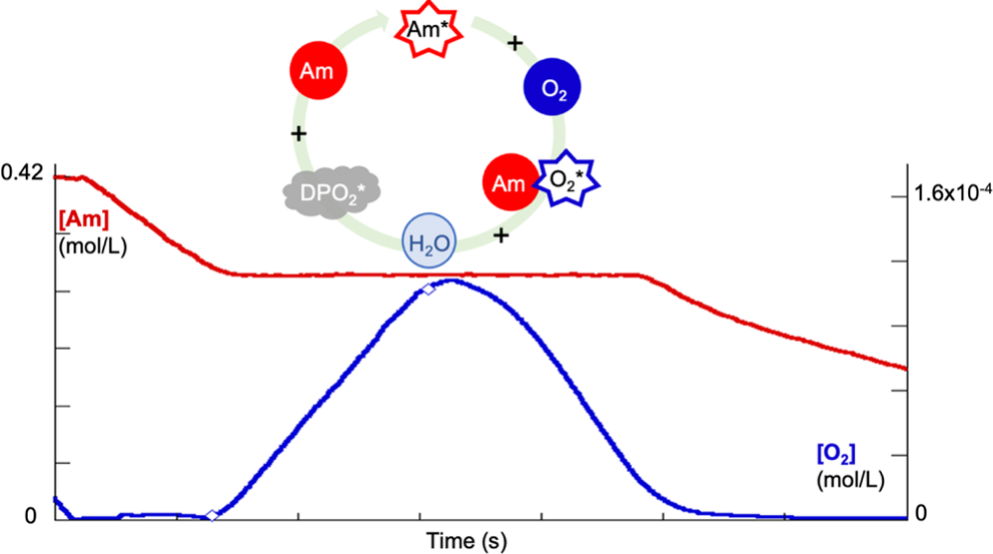

It is known that oxygen (O_2_) stops radical polymerization (RP). Here, it was found that the reaction turn-off occurs abruptly at a threshold concentration of O_2_, [O_2_]_t_, for both free RP and reversible addition–fragmentation chain-transfer polymerization (RAFT). In some reactions, there was a spontaneous re-start of conversion. Three cases were investigated: RP of (i) acrylamide (Am) and (ii) sodium styrene sulfonate (SS) and (iii) Am RAFT polymerization. A controlled flow of O_2_ into the reactor was employed. An abrupt turn-off was observed in all cases, where polymerization stops sharply at [O_2_]_t_ and remains stopped when [O_2_] > [O_2_]_t_. In (i), Am acts as a catalytic radical-transfer agent during conversion plateau, eliminating excess [O_2_], and polymerization spontaneously resumes at [O_2_]_t_. In no reaction, the initiator alone was capable of eliminating O_2_. N_2_ purge was needed to re-start reactions (ii) and (iii). For (i) and (ii), while [O_2_] < [O_2_]_t_, O_2_ acts a chain termination agent, reducing the molecular weight (*M*_w_) and reduced viscosity (RV). O_2_ acts as an inhibitor for [O_2_] > [O_2_]_t_ in all cases. The radical-transfer rates from Am* and SS* to O_2_ are >10,000× higher than the initial chain propagation step rates for Am and SS, which causes [O_2_]_t_ at very low [O_2_].

## 1 Introduction

Due to its triplet ground electronic state, molecular oxygen (O_2_) can act as a radical scavenger by reacting with active radicals and forming derivative-inactive species that can no longer propagate. The presence of O_2_ on polymer chains through the formation of polyperoxides affects the chain structure and length, which can lead to undesirable changes in the final properties of the material.^1^ Not only can O_2_ inhibit conversion completely or delay it by lowering the reaction rates but it can also be exploited to promote reactions. Although many research groups have addressed the role of O_2_ in polymerization reactions, there is still an aura of uncertainty about mechanisms associated with O_2_ and polymerization, and how it can act as both an inhibitor and promoter.^2^

When it comes to polymer manufacturing, free radical polymerization (RP) is one of the most important methods used at the industrial scale, as it generally yields high molecular weight polymers, has low sensitivity to impurities, and is compatible with a wide variety of vinyl monomers and reaction conditions.^3^ However, when it is desired to control specific features of the final product—molecular weight, composition, chain architecture, and so forth, “living” (e.g., anionic polymerization) and controlled RP techniques such as nitroxide-mediated polymerization (NMP), atom-transfer RP (ATRP), and reversible addition–fragmentation chain-transfer polymerization (RAFT) should be employed.^4^ The three above-mentioned reversible-deactivation RP methods are also sensitive to the presence of O_2_ in the reaction medium. However, as is the case for NMP and ATRP, when a catalyst is present, it is preferentially oxidized by the O_2_ molecules, which instead of killing propagating radicals causes a decrease in the amount of catalyst available; this can be easily remedied by using an excess of such compound.^5^ This is not the case for RAFT, which is sensitive to O_2_, as it can bind to intermediate adduct radicals that form during the RAFT mechanism.^6^ Even then, some oxygen-resistant options have been successfully developed.^7^

To avoid unwanted inhibition by the presence of O_2_, reaction media are usually deoxygenated prior to initiation. The two most common methods used are purging with inert gas and carrying out freeze–pump–thaw cycles. Both approaches can be expensive and time-consuming, and in industrial settings, it can be difficult to reach very low O_2_ concentrations in the reaction medium. To overcome this issue, many research groups have developed new techniques toward oxygen-tolerant reactions that mostly account for in situ deoxygenation with enzymes,^8−11^ photosensitizers,^12,13^ photocatalysts,^14,15^ and co-initiators^16^ and, more recently, even by elimination of headspace with an immiscible solvent.^17^

The majority of groups working to address O_2_ effects in reaction kinetics is focused on photopolymerization.^18−20^ There are reports of very small amounts of O_2_ affecting solution RAFT polymerization by permeation through a continuous flow microreactor wall^21^ and of modeling of particle size distribution during emulsion vinyl chloride polymerization,^22^ and kinetic Monte Carlo was used to study autoxidation of polyolefins.^23^ Nonetheless, there are few studies quantitatively investigating how O_2_ affects reaction kinetics, through continuous reaction monitoring, as done in this work.

Most often, the lack of extensive experimental data is the main barrier when it comes to the development of kinetic models. Therefore, techniques that provide continuous reaction data, such as automatic continuous online monitoring of polymerization reactions (ACOMP), are valuable. ACOMP relies on continuous extraction of small reactor content volumes that are appropriately diluted and conditioned to analytical grade for real-time characterization using a multi-detection setup. ACOMP has been extensively reviewed.^24^ Fundamental information about the reaction and the product can be obtained through a variety of detectors—monomer conversion and copolymer composition (cumulative and instantaneous) from ultraviolet (UV) absorption spectroscopy, cumulative and instantaneous reduced and intrinsic viscosity (RV and IV, respectively)^25^ from capillary viscometry, and weight-average molecular weight (*M*_w_) (cumulative and instantaneous) from multi-angle static light scattering (MALS). The detection module can be customized to include other instruments, such as a Fourier transform infrared spectrometer,^26^ a refractive index (RI)^27^ detector, dynamic light scattering, conductivity, polarimetry, and online nuclear magnetic resonance.^28^ The model-free data gathered with ACOMP is what makes it such an invaluable tool for both tracking and modeling reaction kinetics, as well as controlling specific parameters of these reactions. A control interface (CI) coupled to the ACOMP system has been recently developed in collaboration with Fluence Analytics (Stafford, TX). The ACOMP/CI enables fully automatic feedback control of polymerization reactions using a semibatch regime. It has already been employed to produce polymers with specific *M*_w_,^29^ simultaneously controlled composition and *M*_w_,^30^ and multi-modal populations in one pot.^31^

Among the already implemented control variables—feeding of monomers, initiators, and chain-transfer agents—the possible use of a controlled O_2_ feed into the reactor as a reversible chain-shortening agent during RP is introduced here. O_2_ is a potentially interesting control variable as it can be easily removed from the reaction medium by purging with an inert gas, unlike usual chain-transfer agents, such as sodium formate, which cannot easily be removed from the reaction once added. O_2_ could also be used to delay polymerization and suppress gel effects, allowing monomers that tend to autoaccelerate in bulk, like certain acrylates, to viably propagate longer without suddenly reaching extremely high viscosities.^32^ This has been achieved through copolymerization with less reactive monomers.^33^

This work uses controlled O_2_ flow into the reactor and the continuous multi-characteristic data from ACOMP to show the detailed effects of O_2_ on (i) acrylamide (Am) and (ii) sodium styrene sulfonate (SS) during RP and (iii) Am RAFT polymerization. To the best of the authors’ knowledge, it is the first time that an abrupt reaction turn-off due to a critical O_2_ concentration threshold, [O_2_]_t_, has been observed. This threshold phenomenon was found in all reactions, cases (i)–(iii), and was independent of the flow rate of O_2_ into the reactor.

Above [O_2_]_t_, O_2_ acts as an inhibitor, for all the three cases, completely halting the polymerization reaction and leading to a plateau in conversion versus time, during which no monomer is measurably consumed. Below [O_2_]_t_, O_2_ acts as a reversible, inhibitory chain termination agent for RP cases (i) and (ii). During Am RP, case (i), the conversion spontaneously resumes sometime after the O_2_ flow is ceased, regardless of the compressed airflow rate employed. The same is not observed for cases (ii) (SS RP) and (iii) (Am RAFT). For cases (ii) and (iii), the conversion could only be re-started once O_2_ was forcefully purged with nitrogen (N_2_). For case (iii), the “living” type control is maintained after the conversion resumes, following the plateau. This serves as evidence that the RAFT agent itself and its intermediate radicals seem not to be degraded due to sensitivity to O_2_.

It was found in reactions in case (i) that O_2_ is spontaneously eliminated by Am and that the reaction automatically re-starts once the critical O_2_ concentration threshold [O_2_]_t_ is reached. Because there is no evidence of either Am consumption or oligomers/polyoxide formation during the conversion plateau, a cyclic mechanism is proposed here that includes Am radicals acting as a catalytic radical-transfer agent to O_2_, which subsequently reacts, presumably with water, thus removing free O_2_ from the solution. In this process, the O_2_ end product cycles the radical back to Am, which then initiates another radical transfer to O_2_ in the O_2_ elimination step. Once [O_2_] drops below [O_2_]_t_, through this elimination process, the polymerization reaction spontaneously re-starts. A kinetic mechanism was developed that accounts for this proposed radical transfer and zero Am consumption during the conversion plateau. The same critical [O_2_]_t_ seen for Am was also observed during case (ii), RP of SS, whose *M*_w_ and RV were dramatically reduced while [O_2_] < [O_2_]_t_, when compared to analogous reactions carried out under an inert gas atmosphere. These signals approach the non-oxygenated analogous reaction trends after the controlled O_2_ flow is ceased and the monomer conversion was re-established.

It is important to point out that the initiator used, potassium persulfate (KPS), was present in such low concentrations in all reactions that it alone was incapable of eliminating O_2_ detectably in any of the reactions in cases (i)–(iii). If a concentration of KPS is used which is well over an order of magnitude than the highest used in these experiments, then a slow elimination of O_2_ due to the high KPS concentration occurs.

This work is not intended to probe polymerization systems resistant to O_2_, but rather to present detailed experimental results and kinetic modeling concerning the O_2_ threshold phenomenon, in order to stimulate further experimentation and discussion in this area.

## 2 Experimental Section

### 2.1 Materials

Am (99%), SS (≥90%), KPS (≥99%), and sodium phosphate dibasic (≥99%) were purchased from Sigma-Aldrich. The RAFT chain-transfer agent (CTA) 4-((((2-carboxyethyl)thio)carbonothioyl)thio)-4-cyanopentanoic acid (BM1433, ≥95%) was acquired from Boron Molecular. The half-life of KPS in water at 60 °C is around 10 h and 34 h at 50 °C. Compressed air was used as the O_2_ source. All materials were used as received. Table 1 shows the structures of the two monomers and the RAFT agent employed.

**Table 1:**
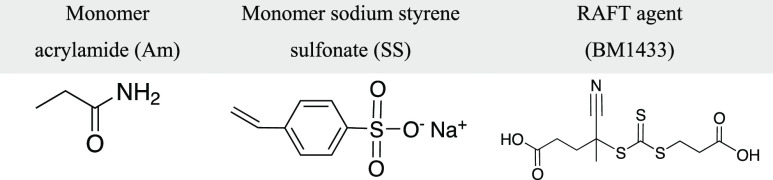
Chemical Structures of Monomers and CTA

### 2.2 Reaction Conditions

Deionized water was used as the solvent for all reactions with Am, and a 100 mM sodium chloride (NaCl) solution was used for reactions with SS to suppress polyelectrolyte effects. RP and RAFT reactions had identical conditions, unless stated otherwise. This provided the basis for comparison between reaction kinetics using RP and RAFT when exposed to O_2_. For the RAFT reactions, where a *M*_w_ target of 30,000 g/mol was used, sodium phosphate dibasic was added to the initial reactor solution to neutralize the carboxylic acid groups in BM1433 and keep pH around 5 to help its solubilization and inhibit its degradation throughout the reaction. Prior to addition to the reactor, KPS was diluted in 2 mL of solvent. The reactor contents were purged with ultra-high-purity N_2_ for 40 min before initiator addition up to some initial period into the reaction.

A compressed air source was employed to flow O_2_ into the reaction medium at varying rates, from 6 to 1000 sccm (standard cm^3^/min). After a certain O_2_ flow period, the flow was stopped and monitoring was continued. For reactions at 50 °C, the dissolved O_2_ content in g/cm^3^ was measured with an in situ rugged dissolved oxygen (RDO) probe connected to a Thermo Fisher Orion Star A216 pH/RDO/DO meter, along with ACOMP monitoring. The RDO probe employed has a resolution of 0.01 × 10^–6^ g/cm^3^ and a relative accuracy of 0.1 × 10^–6^ g/cm^3^. Reactions at 65 °C were above the maximum operating temperature of the O_2_ probe.

Several control experiments were carried out: analogous reactions with no O_2_ addition and monitoring each of the reactants and product individually when exposed to O_2_, outside a polymerization reaction. Table 2 lists the specific conditions for all reactions. Detailed oxygenated conditions for all reactions are presented in Table S1 as Supporting Information. Specific experimental conditions for all other control experiments are included in Tables S2 and S3. Control experiments were all carried out at 50 °C to enable the use of the RDO probe. The reactor was vented to atmosphere via a narrow bore needle in a septum on one of the reactor necks, during all experiments. After the system was purged thoroughly with N_2_ and allowed to sit, no measurable O_2_ flowed back through the needle into the reactor over the period of a day.

**Table 2:** List of Reactions with Initial Conditions

reaction (case)	O_2_ flow rate (sccm)	type	temperature (°C)	monomer	[monomer] (mol/L) ×10^1^	[KPS] (mol/L) ×10^3^	[BM1433] (mol/L) ×10^3^
1A (i)	0	RP	65	Am	4.22	1.66	–
2A (i)	15	RP	65	Am	4.22	1.67	–
3A (i)	75	RP	65	Am	4.22	1.66	–
4A (i)	192	RP	65	Am	4.22	1.67	–
5A (i)	1000	RP	65	Am	4.22	1.67	–
6A (i)	200	RP	50	Am	4.78	3.77	–
7A (i)	200	RP	50	Am	4.78	3.77	–
8A (i)	200	RP	50	Am	4.22	1.66	–
9A (i)	200	RP	50	Am	4.22	3.33	–
10A (i)	200	RP	50	Am	4.22	3.33	–
11A (i)	1000	RP	50	Am	2.11	6.66	–
12A (i)	6	RP	50	Am	4.22	3.33	–
13A (i)	0	RP	50	Am	4.22	3.33	–
1B (ii)	6	RP	50	SS	1.43	3.33	–
2B (ii)	0	RP	50	SS	1.43	3.33	–
1C (iii)	15	RAFT	50	Am	7.03	0.337	1.69
2C (iii)	15	RAFT	50	Am	7.03	0.337	1.69

### 2.3 Automatic Continuous Online Monitoring of Polymerization Reactions

All reactions were carried out using a Fluence Analytics (Stafford, Texas) third generation fully automated laboratory ACOMP system, equipped with a 500 mL reactor. The detector train included a UV detector (Testa Analytical, Berlin, Germany), a Fluence MALS unit, and a single capillary viscometer. A full experimental setup is provided in the Supporting Information, as Figure S1, following a description of the ACOMP dilution scheme and use of detectors.

### 2.4 Gel Permeation Chromatography

Aliquots were taken from the ACOMP waste stream during reactions for offline gel permeation chromatography (GPC) analysis during reactions. This served not only as a complementary cross-check to the ACOMP conversion data but also as a means to identify any polymer formation during the conversion plateau period (no evidence for polymer formation on the conversion plateau was found, as discussed below). The custom-built GPC system is described in detail in the Supporting Information in Section SI1.

## 3 Results and Discussion

### 3.1 Initial Considerations on Oxygen in the Reactor Solution versus Headspace

Before presenting the data from the polymerization reactions, it is important to discuss O_2_ dissolution and saturation in the reactor solution and in the headspace to better understand underlying flows from the latter to the former and how they affect the reaction results. While flowing O_2_ (air) into the reactor over a defined interval of time is straightforward, there are some subtleties on how O_2_ splits between the reactor headspace and reactor liquid. Salient features include the fact that the concentration of O_2_ in the headspace at 50 °C is 58× greater than O_2_ dissolved in water (see Table S4, under Section S2), and, in the specific system used, saturation of the headspace by O_2_ was much more rapid than in the reactor liquid. This means that if the airflow into the reactor is cut off before the liquid is saturated with O_2_, there will be a backflow of O_2_ from the headspace into the liquid. The effect of O_2_ backfill into the reactor solution is very prominent during RAFT reactions and helped to unravel the O_2_ mechanism in RP and discern the difference in O_2_ effects between RP and RAFT, as discussed below. A more in-depth discussion and results concerning O_2_ in the reactor solution versus the headspace are given in Section S2 of the Supporting Information.

A few relevant parameters pertaining to this process can be easily calculated. For the reactor initially purged thoroughly with N_2_, with subsequent controlled inflow of air into the liquid, [O_2_] followed the expected first-order build-up of the form in both water and headspace
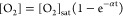
1where [O_2_]_sat_ is the saturation concentration of O_2_ and α is the O_2_ rise rate in s^–1^ in either air or water. Given ideal mixing, the expected form of α is simply
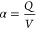
2where *Q* is the volume flow rate of O_2_ into the reactor and *V* is the volume of either the headspace or the reaction liquid. The α values found for the headspace, according to eq 2, are very close to the ones obtained by using eq 1. However, for the reactor liquid, the O_2_ rise values from the exponential fit (eq 1) of the data were found to be significantly smaller than those predicted by eq 2, presumably due to the details of gas bubbles diffusing into the liquid, which is a function of multiple variables, such as mixing, the solvent itself, and temperature, thus causing the deviation from ideal mixing. These results are compiled in Figure S2 in Section S2 under O_2_ in Water. The significant difference in O_2_ rise and saturation found when comparing the headspace to the reactor liquid is even more pronounced during a reaction and can also be observed during monitoring of the initial O_2_ purge with N_2_ (see Figure S4 in Section S2 under Reaction O_2_ Monitoring). These features of O_2_ diffusion and solubility in solution are important for analyzing the polymerization experiments, presented next.

### 3.2 Assessment of Abrupt Reaction Turn-Off during Am RP (Case (i))

A feature common to all reactions for cases (i)–(iii) was found, an abrupt turn-off of the reaction as O_2_ flowed into the reactor. The turn-off occurs abruptly when a certain threshold concentration of oxygen, [O_2_]_t_, is reached. While it is well known that O_2_ halts RP reactions, the approach to the turn-off could be, a priori, gradual rather than abrupt. The authors could not find any literature demonstrating the threshold nature of the O_2_-induced turn-off. This feature, common to all reactions in all the three cases, was independent of the O_2_ flow rate into the reactor, which was tested from 15 to 1000 sccm for case (i).

**Figure 1:**
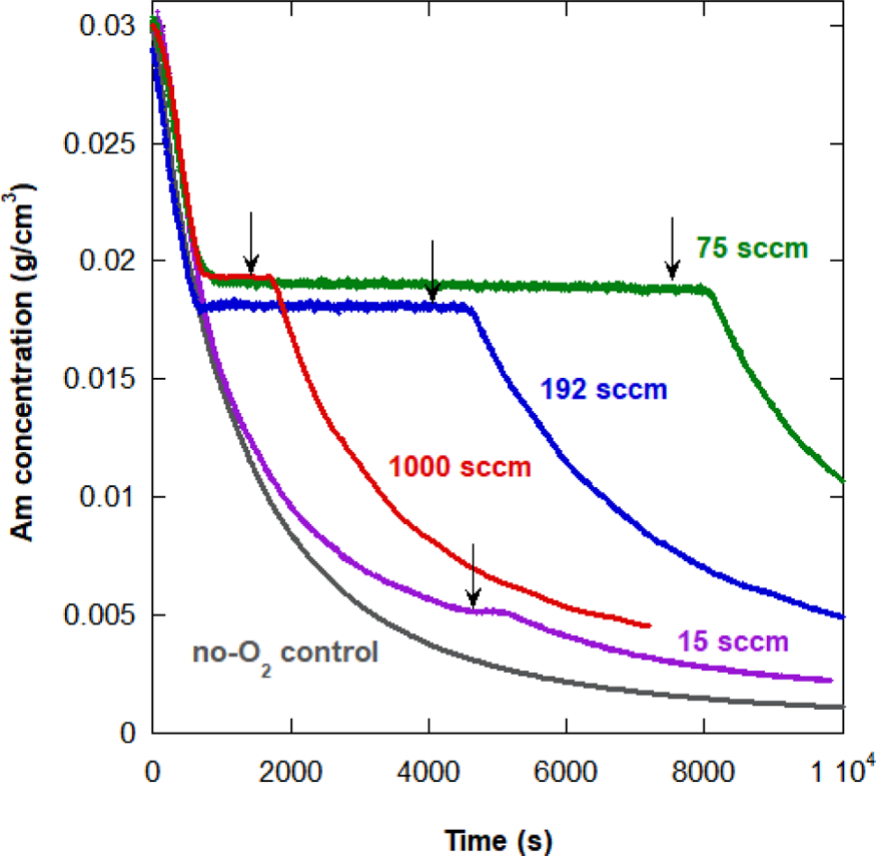
Am concentration in the reactor, showing conversion plateaus obtained with several flow rates during Am polymerization reactions 2A–5A in Table 2, followed by spontaneous re-start after the end of the compressed airflow (black arrows). The control no-O_2_ reaction is 1A.

Figure 1 shows Am conversion curves versus time for case (i) reactions 2A–5A in Table 2, in which the flow rate of air into the reactor varied from 15 to 1000 sccm. In Figure 1, the conversion is given in terms of the remaining Am concentration, [Am], in the reactor. Reaction 1A served as a control, in which there was no O_2_ introduced into the reactor; that is, it underwent constant purging with N_2_ as is usually done in RP. In all oxygenated cases, reactions 2A–5A, there is an abrupt reaction turn-off, a plateau in which no measurable conversion occurs, and an abrupt, *spontaneous* re-start of the reaction. Also shown in Figure 1 is the point at which the airflow was stopped (black arrows); all flow rates started at the same time; *t* = 300 s. It is seen in each case, reactions 2A–5A, that there is a delay between the airflow stoppage and the spontaneous turn-on of the reaction. The delay corresponds to what is hypothesized to be the catalytic elimination of O_2_ by Am radical, which is modeled below.

**Figure 2:**
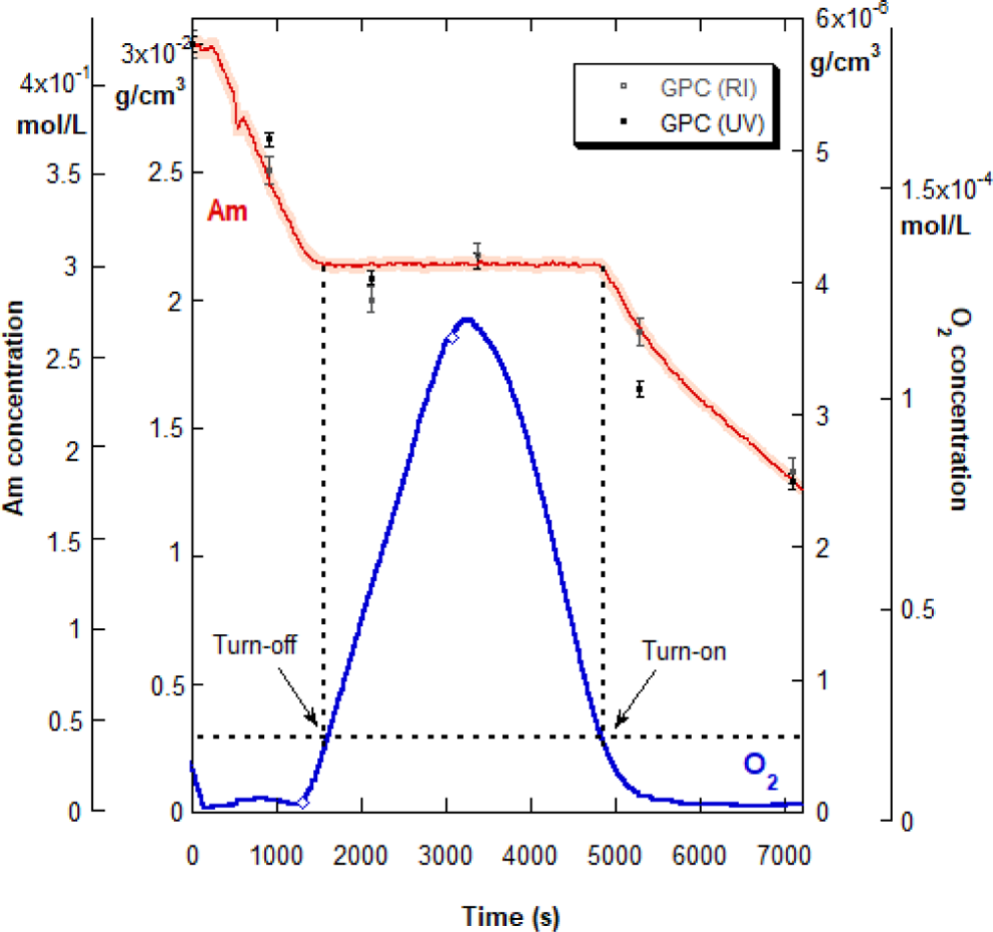
Simultaneous reactor measurement of dissolved [O_2_] in solution and [Am] for reaction 8A in Table 2, case (i), showing the abrupt turn-off and spontaneous re-start events. The diamonds on the O_2_ curve mark the beginning and the end of the compressed airflow period. The shaded area around [Am] data shows the error range of [Am], as measured by ACOMP. Also shown are discrete points, with error bars, corresponding to [Am] calculated from offline GPC analyses of aliquots taken during the reaction.

To better understand the behavior of O_2_ during the reactions, the reaction temperature was dropped from 65 °C (above) to 50 °C so that the RDO probe could be employed to measure dissolved O_2_. Figure 2 shows the time course of both Am and O_2_ concentrations for a typical reaction (8A, in Table 2). The two diamonds on the O_2_ curve show the beginning and end of the airflow period. In most RP reactions, there was a slight rise in [O_2_] after the airflow is stopped due to the O_2_ backflow from the headspace, as discussed in the above section. However, the O_2_ elimination process, modeled kinetically below, quickly overwhelms the headspace backflow of O_2_ and [O_2_] drops to the critical threshold [O_2_]_t_, at which point the reaction abruptly re-starts. The authors could find no literature reports of the spontaneous re-start of an oxygen-halted RP. The simultaneous monitoring of reaction kinetics and the O_2_ in the solution allowed the connection to be made between the observed abrupt turn-off with [O_2_] when [O_2_]_t_ is reached with O_2_ inflow and the spontaneous re-start when [O_2_] drops below [O_2_]_t_ on the conversion plateau.

Also shown in Figure 2 is the error bar range on the Am concentration calculated by ACOMP, which is around 1%. Within the error bars, seen as the shaded portion of the [Am] curve, there is no measurable change in the Am concentration on the conversion plateau in any of the reactions. A closer look at possible Am consumption during the plateau is considered below. The measurable change needed for a one-to-one elimination of O_2_ by Am was never found, indicating that Am is not used up in the O_2_ elimination process but is necessary for it to occur—as shown by the various control experiments below; Am acts as a catalyst, driving the O_2_ elimination reaction without being measurably consumed. A scheme for this inferred process is provided in the Kinetic Model section.

As a further cross-check on the ACOMP data, offline GPC analyses of aliquots taken at different time points in the reaction were performed and can be seen in Figure 2 as the discrete points for [Am]. Error bars are the standard deviation from multiple injections of the same sample. Based on both UV and RI signals, there is good agreement with the continuous ACOMP data. The more scattered GPC data is likely due to the errors related to manual dilution prior to analyses and instabilities or drift on the signal baselines, which affect all further calculations.

#### 3.2.1 Control Experiments Show Spontaneous O_2_ Elimination in Specific Circumstances

**Figure 3:**
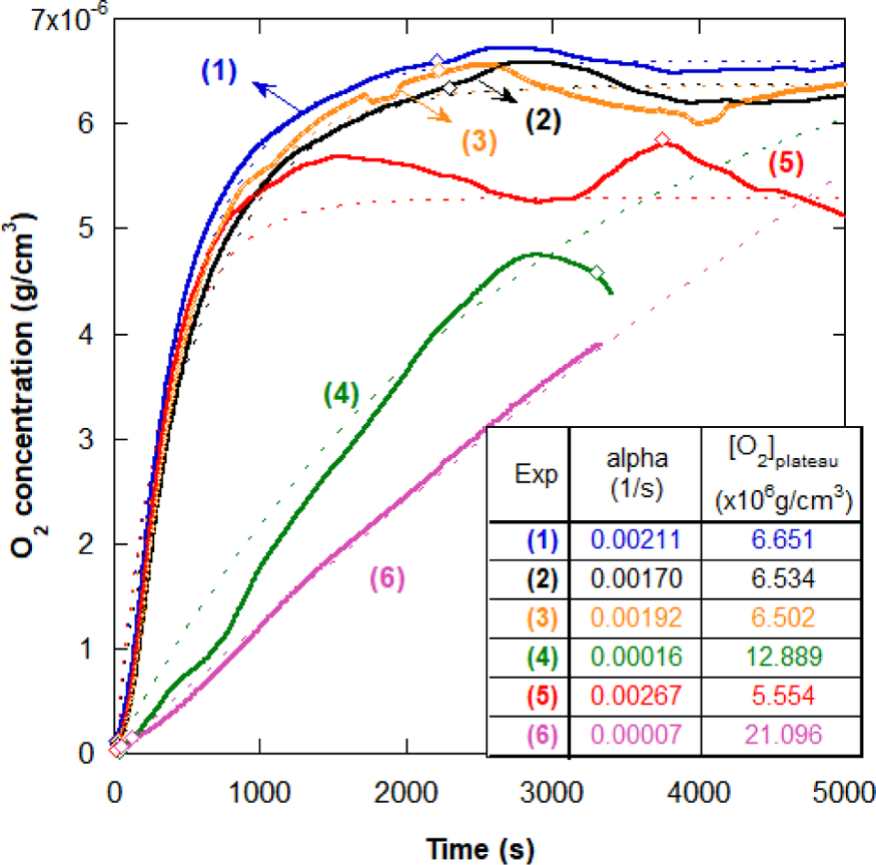
Control experiments showing O_2_ concentration in solution for (1) pure water, (2) KPS in water at 3.77 × 10^–3^ mol/L, (3) Am in water at 0.478 mol/L, (4) a reaction with the same concentrations of KPS and Am as (2) and (3) (7A in Table 2), (5) a final reactor content (98% conversion, pAm in water from reaction 6A in Table 2), and (6) pure water solely due to the backflow from the headspace. The diamonds in each curve mark the beginning and the end of the compressed airflow period. The values of α and [O_2_]_plateau_ in the table were obtained according to eq 1.

Figure 3 shows a collection of important control experiments: dissolved O_2_ versus time with a flow rate of 200 sccm air into the reactor at 50 °C in (1) pure water, (2) water with KPS at 3.77 × 10^–3^ mol/L, (3) water with Am at 0.478 mol/L, (4) a reaction with the same concentrations of KPS and Am as (2) and (3), and (5) a final reactor content [98% conversion; polyacrylamide (pAm) in water] from a reaction with the same conditions as (4). For (6), the system was oxygenated at 15 sccm for a very short flow period, with virtually all the O_2_ quickly going into the headspace; the subsequent O_2_ increase seen in the figure is due purely to the backflow from the headspace, after the airflow was shut off. Detailed experimental conditions are provided in Tables S2 and S3 of the Supporting Information. The rise rates α from eq 1 are shown in the box in Figure 3. For (1)–(3), the rise rates are comparable with ⟨α⟩ = 0.0019 ± 0.0001 1/s, whereas they are much slower for (4) and (6). (1)–(3) reach comparable values of saturation averaged at 6.56 × 10^–6^ g/cm^3^ ± 0.045 × 10^–6^.

With the exception of experiment (6), all control experiments were carried out using a 200 sccm O_2_ flow rate that was kept until a saturation plateau was achieved and then shut off. All experiments were initially purged with N_2_ until approximately 5 min after time zero (*t* = 0), set to align with the initiator addition for the reaction (4 in Figure 3).

Importantly, Figure 3 shows that Am alone has no effect on [O_2_] and that for KPS alone, at the concentration used in most reactions, there is also no noticeable change in the O_2_ profile and saturation level; that is, radicals I* formed from KPS decomposition alone are not sufficient to “scavenge” the O_2_ at the order of magnitude of the concentrations used in the reactions and that any radical O_2_^*^ formed by transfer from I* does not propagate significantly to other O_2_. The dramatically lower value of O_2_ during the reaction (and slope) shows the synergy that occurs when all reactants are together during RP and cause elimination of O_2_; that is, Am has a decisive role and yet is not measurably consumed as O_2_ is eliminated during the conversion plateau.

The final reactor content (5 in Figure 3) is saturated at lower values than pure water, KPS in water, and Am in water and shows fluctuations rather than a well-defined plateau. The fluctuations are possibly due to the high viscosity of the polymer solution, mixing effects, and O_2_ dissolution in water, so its concentration might not have been completely homogeneous within the reactor, adding a source of error to the measurements. For (5) as well, there is a slight decrease in [O_2_] after the airflow period, which may be related not only to mixing effects but also to O_2_ being released to the headspace through mixing of the solution and then going back into solution, until the system reaches an equilibrium [O_2_]_sat_ between 5.56 and 6.65 × 10^–6^ g/cm^3^. The lower [O_2_]_sat_, when compared to (1)–(3), may be due to residual Am in the final product eliminating some O_2_. The slow rise rate of [O_2_] for (4), compared to the fast rate in (5), shows the strong effect the O_2_ elimination cycle has on [O_2_] and its rate of change.

Experiment (6) was designed so that the increase in [O_2_] in the reactor liquid due purely to headspace backflow could be measured. The backflow rate of O_2_ for Experiment (6) is higher than that calculated from an overnight experiment designed to track the backflow from headspace when the only source of O_2_ is the venting needle, whose α = 1.9 × 10^–5^ 1/s. This makes sense since the rate of backflow is proportional to the headspace volume and its [O_2_]. In the kinetic model, this will introduce an approximately constant source term for O_2_ after the airflow stops.

As can be seen through the dip in [O_2_] even before the end of the airflow period in (4), if O_2_ is flowed long enough to reach a steady concentration, in case (i), the concentration plateau is below the O_2_ saturation level in pure water. The plateau height scales with increasing plateau [Am] (not shown), which likely relates to the number of Am* available to scavenge O_2_ molecules while compressed air is pumped into the reactor.

It is not expected that the presence of any of the reagents, at the concentrations used, would affect the solubility limit of O_2_ in the reactor, and no difference in solubility limits was found, within error bars, for the presence of each reagent alone. The purpose of these control experiments was to show that the *rate* of dissolved O_2_ in the reactor solution is unaffected by the presence of each reagent in isolation but is dramatically slowed when a reaction occurs and Am is present.

In order to assess if, at much higher concentration, primary radicals from KPS would also be able to scavenge O_2_, two identical experiments were carried out using KPS in water at different concentrations—3.78 × 10^–3^ mol/L (the highest used for RP reactions) and 50× higher at 1.85 × 10^–1^ mol/L. Results are shown in Figure S3. As expected, at much higher concentrations, KPS radicals are capable of consuming the O_2_ injected into the aqueous medium. However, the O_2_ consumption at high [KPS] is exponential, indicating a simple first-order reaction of the type, , where γ is the decay rate. In contrast, the O_2_ elimination shape in Figure 2 is more complex than a simple exponential, and the rate of elimination here is much slower than with Am during a reaction. In the kinetic model, this will introduce another O_2_ loss term after the airflow stops. ***The experiments compiled in***Figure S3***show that the contribution to O***_***2***_***elimination from KPS is negligible at all the [KPS] used in the polymerization reactions.*** For case (i), there are not enough free radicals produced by the initiator to individually destroy the number of O_2_ molecules present. In fact, the amount of O_2_ present is typically more than the total amount of KPS, let alone the small amount of KPS decomposing into radicals during the conversion plateau.

#### 3.2.2 No Evidence of Polymer Formation during the Conversion Plateau

**Figure 4:**
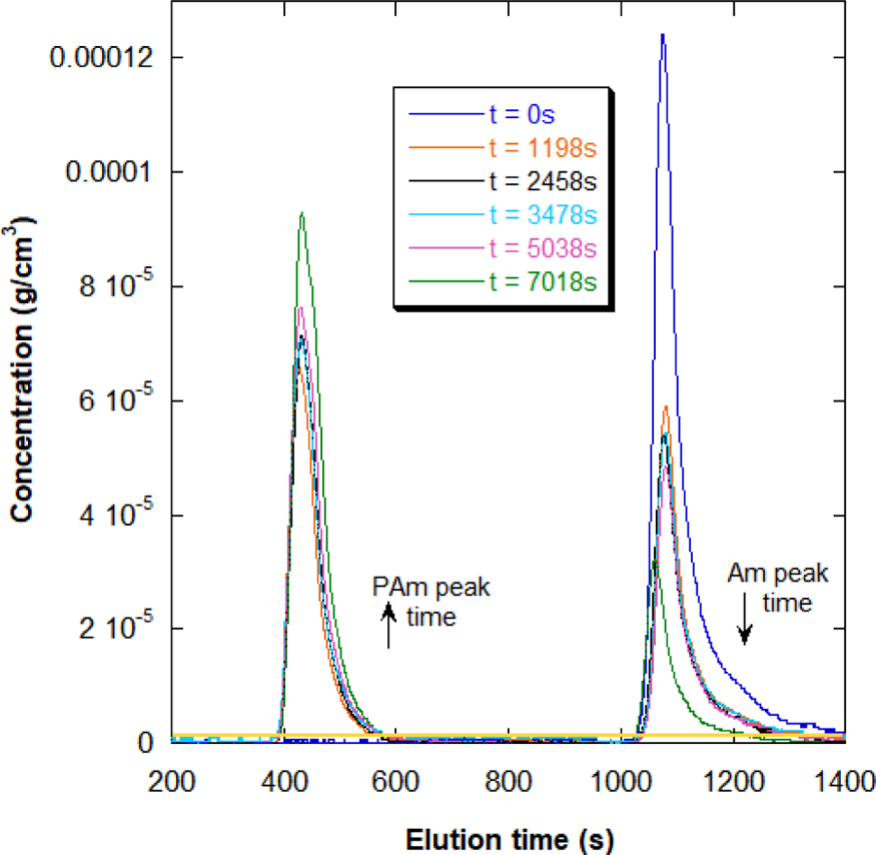
Elution chromatograms from GPC analyses of aliquots extracted from reaction 9A in Table 2. The yellow line is set at 5 standard deviations, and it represents the minimum polymer concentration detectable, which is 1.5 × 10^–6^ g/cm^3^. The conversion plateau occurred between reaction time 2000–4000 s.

Offline GPC analyses of aliquots extracted at different times during the reactions were also analyzed to assess whether other polymer chains with intermediate to low molecular weight, or oligomers, were produced during the observed conversion plateaus. Figure 4 shows the concentration of Am and its polymer (pAm) calculated from the RI data using the d*n*/d*c* of each species. As indicated in the plot, the Am peak shows up after 1000 s of elution, and the pAm peak starts at around 400 s. The minimum polymer concentration detectable, determined from 5× the standard deviation of the GPC baseline, was 1.5 × 10^–6^ g/cm^3^ and is shown as the horizontal yellow line in Figure 4. There is no signal in the above-said line, which indicates that no detectable intermediate products were obtained during the conversion plateau, that is, ***no detectable polymerization into possible polyoxides occurred and no Am consumption could be detected***. Yet O_2_ was vigorously consumed during the conversion plateau, suggesting a catalytic effect due to Am, since KPS is not abundant enough for the elimination and no measurable amount of Am is consumed. Spontaneous formation of poly(acrylamide peroxide) has, however, been previously reported in very specific circumstances that are widely different from those employed here.^34^

### 3.3 Investigation of O_2_ Effects in SS RP (Case (ii)) and Am RAFT Polymerization (Case (iii))

**Figure 5:**
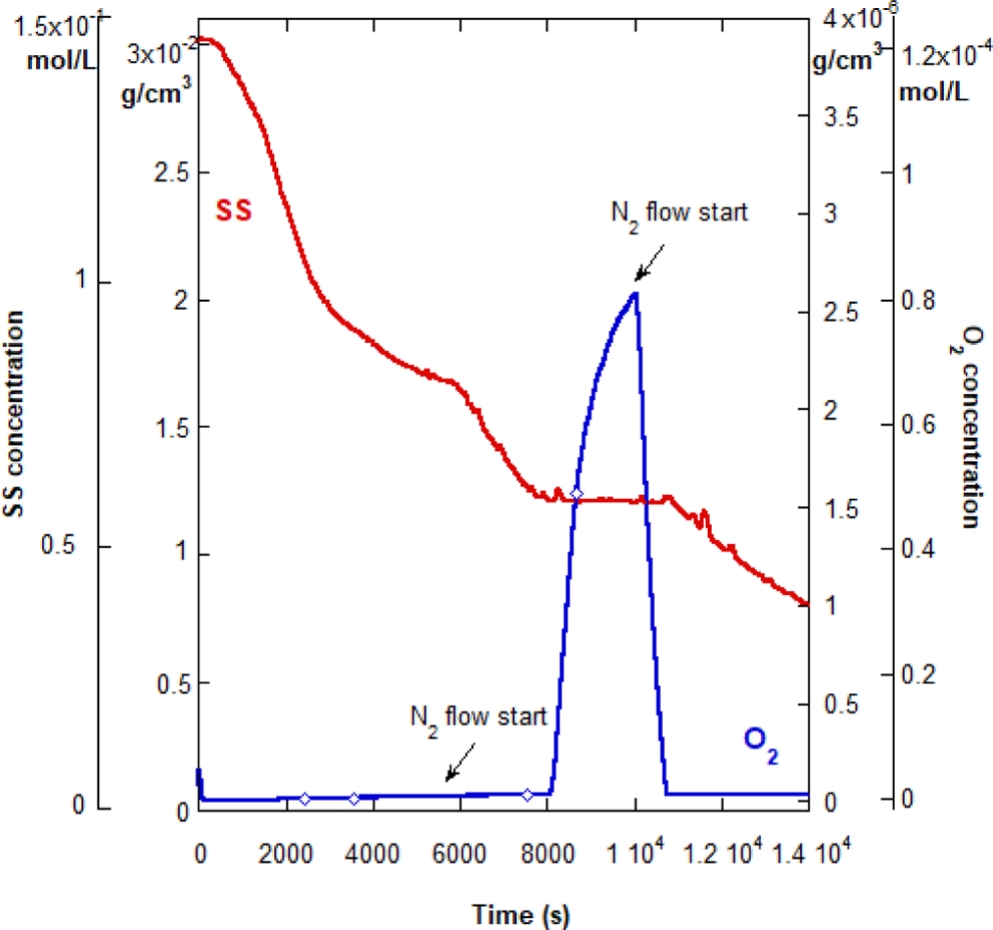
Simultaneous reactor measurement of dissolved O_2_ in solution and SS concentration for reaction 1B in Table 2, case (ii), showing the abrupt turn-off and reaction plateau. Unlike Am RP, O_2_ is not eliminated on the plateau. The strong subsequent rise in O_2_ is due to the backflow from the headspace. The reaction was forcefully re-started by purging with N_2_, as indicated with arrows. The diamonds on the O_2_ curve mark the beginning and the end of the compressed airflow period.

Continuing with RP reactions, another monomer was used for further investigation of the abrupt turn-off. Case (ii) involved RP of SS. Figure 5 shows the abrupt turn-off threshold, but in this case, there is no drop in [O_2_] on the plateau nor is there a spontaneous reaction re-start. Re-start occurs only by purging the excess O_2_ with N_2_. As shown in Figure 5, once the compressed airflow is shut-off, [O_2_] increases due to the backflow from the headspace. Fitting the [O_2_] via eq 1, [O_2_]_sat_ = 2.7 × 10^–6^ g/cm^3^ is obtained, which is much lower than the values obtained from the control experiments. The calculated O_2_ rise rate at 0.015 s^–1^, however, is comparable to all control experiments not involving a polymerization reaction. This is indicative that there is not a substantial presence of radicals scavenging O_2_, to cause a decrease in α, as found for case (i), or that, as further surmised, SS* has an energy level closer to that of O_2_ and is not as likely as Am* to transfer their radicals to O_2_. The difference in the [O_2_] curve once compressed air is shut off, compared to Am RP, is stark; for case (i), there is an immediate measurable decrease in [O_2_], which is not seen here. Spontaneous re-start after a much longer time, even though not observed here during the monitoring period, cannot be ruled out. It is, however, improbable as the data points to a slow rate of radical transfer from SS* to O_2_, which leads to an O_2_ consumption which is less than the O_2_ backflow from the headspace.

**Figure 6:**
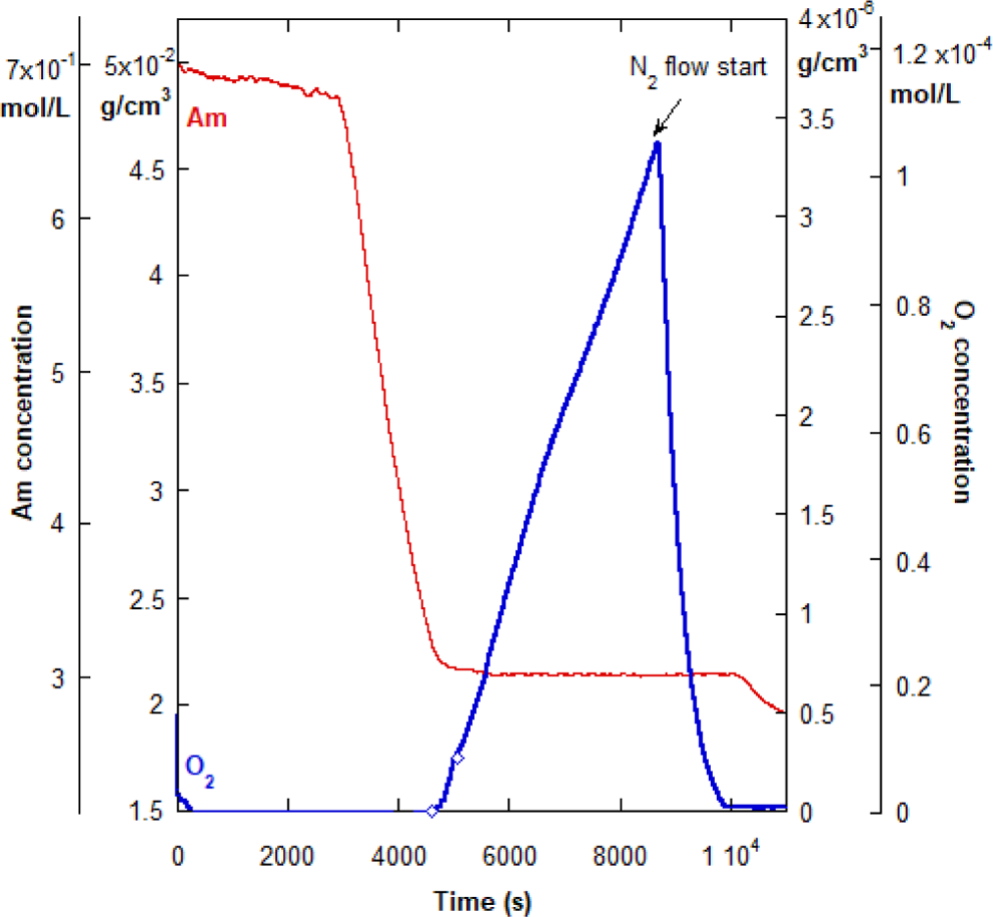
Abrupt turn-off threshold for RAFT reaction 1C in Table 2. Unlike Am RP, O_2_ is not eliminated on the plateau. The reaction was re-started by purging the reactor with N_2_, just like in case (ii). The strong, subsequent rise in O_2_ is due to the backflow from the headspace. The diamonds on the O_2_ curve mark the beginning and the end of the compressed airflow period.

Since the reaction turn-off was consistent for all reactions in cases (i) and (ii) for RP of Am and SS, respectively, it was of interest to know what would happen when a controlled polymerization technique, in this case, RAFT, was used. Figure 6 shows that there is a sharp threshold shut-off of the Am conversion, just as for RP cases (i) and (ii). However, [O_2_] does not decrease once the compressed airflow stops, which also occurred in case (ii). In fact, [O_2_] increases considerably in Figure 6 due to the backfilling from the headspace, which is consistent with the pure water control in Figure 3 (experiment 6). Therefore, there seems to be little to no O_2_ scavenging by Am*, as was observed for RP reactions. The initial delay observed in Figure 6 is due to the initial RAFT equilibrium, which becomes slower as the temperature decreases (e.g., from 65 to 50 °C).

It was only possible to re-start the RAFT reaction while on the conversion plateau by purging the reactor with N_2_. As can be seen in Figure 6, conversion resumed at around *t* = 10,000 s. Again, the strong increase in O_2_ due to the backflow from the headspace, after the airflow stops, can be observed as well. Extrapolating [O_2_] via eq 1 yields a final [O_2_]_sat_ value of roughly 6 × 10^–6^ g/cm^3^, which is at or near saturation in water.

**Figure 7:**
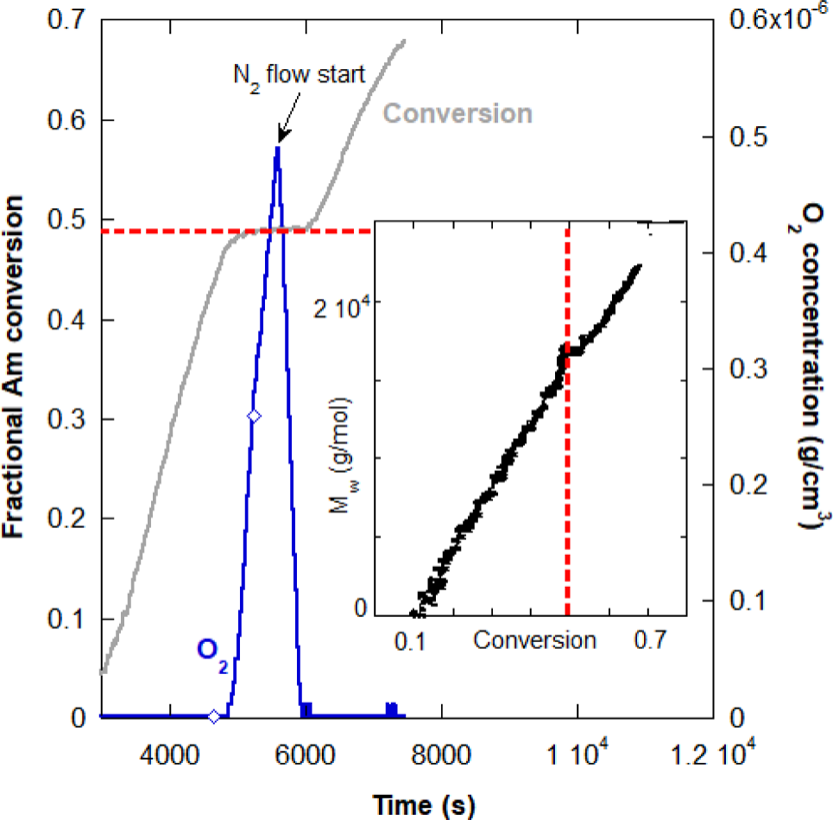
Abrupt turn-off threshold for RAFT reaction 2C in Table 2. The reaction was re-started by purging the reactor with N_2_. The inset shows that reaction control was maintained after the re-start of conversion. The horizontal dashed red line highlights the conversion plateau in the main plot and corresponds to the vertical, red dashed line in the inset. The diamonds on the O_2_ curve mark the beginning and the end of the compressed airflow period.

Proof that the re-started reaction is still controlled, and not RP, is given in Figure 7, which shows another RAFT reaction, 2C in Table 2. It was stopped by airflow, leading to a fractional Am conversion plateau at around 0.5. The inset to the figure shows the linear increase of *M*_w_ with conversion, as expected for RAFT and other living-type reactions. The vertical, dashed red line shows where the reaction was re-started due to the N_2_ purge. The inset shows that control was immediately regained when the reaction re-started, and *M*_w_ continued to increase linearly with conversion.

While cases (ii) and (iii) showed the same abrupt turn-off and conversion plateaus as case (i), neither of them eliminated O_2_ on the conversion plateau, so that there was no spontaneous re-start in either case.

### 3.4 O_2_ as a Chain Termination Agent below the O_2_ Threshold in RP

In all the three cases, (i)–(iii), at a certain threshold concentration, [O_2_]_t_, the reaction abruptly stops and remains stopped as long as air continues to flow. The exact and reproducible [O_2_]_t_ values, however, could not be obtained due to mixing issues and the resolution of the O_2_ probe, but they were always lower than 0.6 × 10^–6^ g/cm^3^ (1.88 × 10^–5^ mol/L), often substantially below this, as can be seen in Figures 5 and 6.

**Figure 8:**
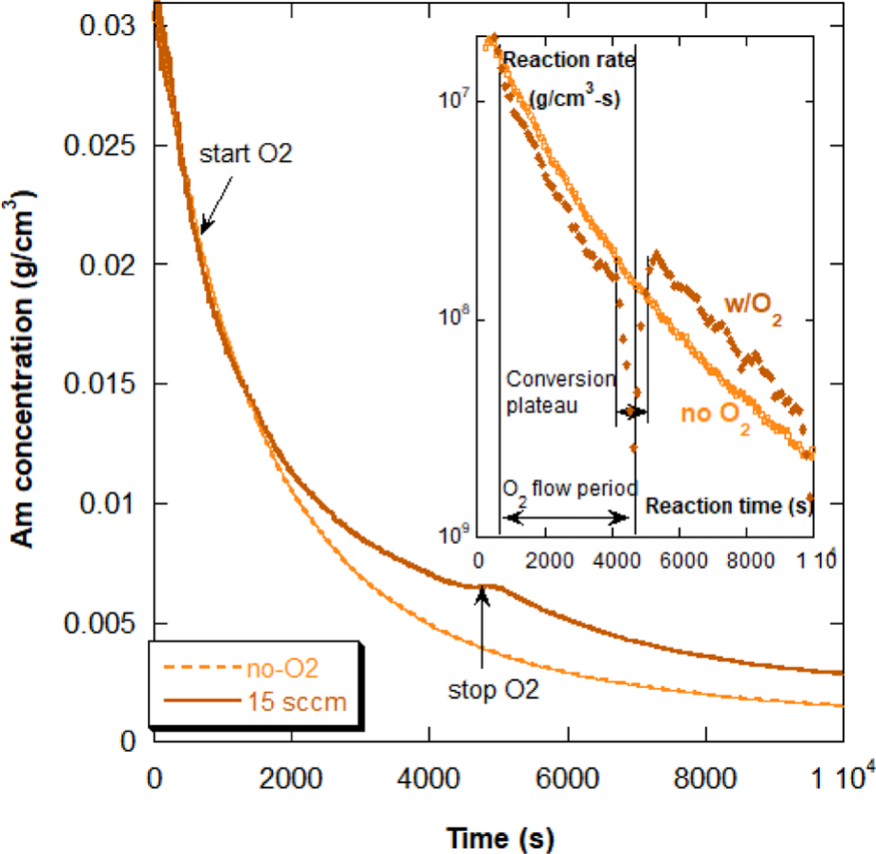
Conversion of Am with no airflow and compressed airflow rate at 15 sccm (reactions 1A and 2A in Table 2, respectively). The inset shows the reaction rate of the lowest airflow reaction (interrupted curve) and of an N_2_ purged reaction with no airflow (uninterrupted curve).

While O_2_ completely inhibits the polymerization reactions above [O_2_]_t_, it was of interest to explore how reaction kinetics were affected for [O_2_] < [O_2_]_t_. In short, the reaction is slowed by O_2_ and polymer chains are shortened, compared to the O_2_-free reactions. Figure 8 shows how the reaction is measurably slowed as soon as air begins to flow into the reactor, as also observed in Figure 5 during the first compressed airflow period. The turn-off and re-start thresholds in Figure 8 are seen at 4700 and 5200 s, respectively. The inset in Figure 8 shows the rates of the reaction for the data with the 15 sccm airflow and for an identical reaction in which there was no airflow (reactions 2A and 1A in Table 2, respectively). The rate with the airflow drops abruptly at the threshold [O_2_]_t_ and is measurably lower than for the no-O_2_ reaction, and the rate recovery is seen when the conversion plateau ends, and the reaction re-starts spontaneously. In fact, displacing the reaction rate after the re-start back to the beginning of the plateau (not shown, but can be seen visually) recovers the rate of the reaction for the O_2_ free reaction; that is, the initial airflow and the plateau have had no effect on Am to subsequently polymerize, after O_2_ elimination, just as it would have polymerized had there been no airflow and conversion plateau. This is similar to the recovery of control during case (iii), RAFT Am polymerization, in which the controlled reaction proceeded after N_2_ purge as if the reaction had never been initially stopped by O_2_.

Given the continuous nature of ACOMP measurements, it is possible to take derivatives along the reaction and obtain instantaneous values from cumulative properties such as *M*_w_ and RV. The instantaneous weight-average molecular weight, *M*_w,inst_, is computed for RP from the cumulative *M*_w_ and the polymer concentration *C*_p_ according to
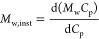
3a

The instantaneous reduced viscosity RV_inst_ is obtained from the cumulative reduced viscosity, RV, and *C*_p_ in a similar way
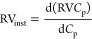
3b

**Figure 9:**
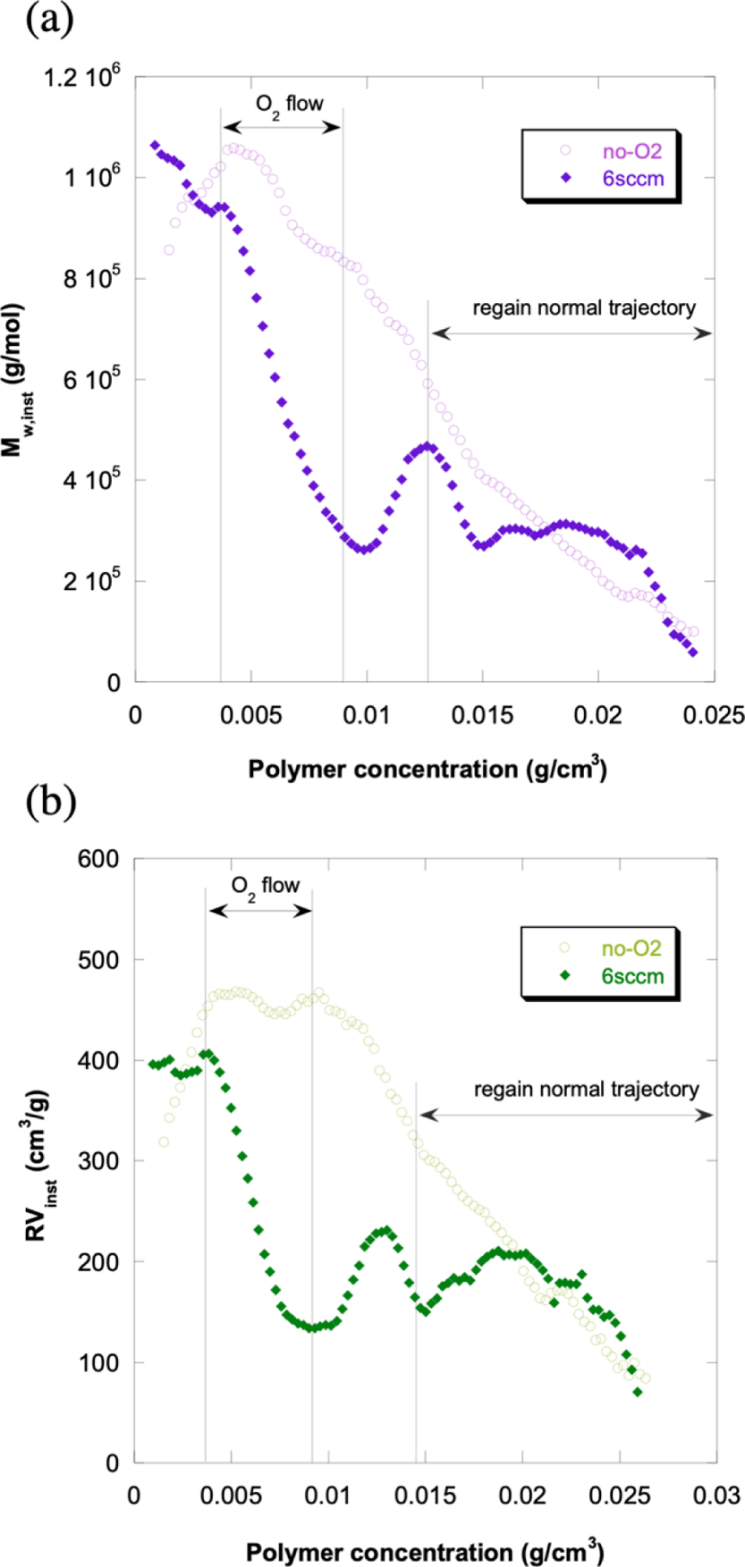
(a) Instantaneous weight-average molecular weight (*M*_w,inst_) for polymerization of Am with 6 sccm airflow and with no O_2_ (reactions 12A and 13A in Table 2, respectively). The effect of O_2_ as a chain termination agent, decreasing *M*_w,inst_, is very large. Once the airflow is ceased, the reaction resumes after automatic O_2_ elimination, and *M*_w,inst_ for 6 sccm rejoins the values for the reaction without O_2_. (b) Instantaneous reduced viscosity (RV_inst_) for polymerization of Am with 6 sccm airflow and with no O_2_ (reactions 12A and 13A in Table 2, respectively). The effect of O_2_ as a chain termination agent, decreasing RV_inst_, is very large and similar to the behavior of *M*_w,inst_. Once the airflow is ceased, the reaction resumes after automatic O_2_ elimination, and RV_inst_ for 6 sccm rejoins the values for the reaction without O_2_.

Figure 9a compares *M*_w,inst_ for a reaction where O_2_ was injected at 6 sccm and for an identical reaction with no O_2_ flow (reactions 12A and 13A in Table 2, respectively). Figure S5 shows the cumulative weight-average molecular weight, *M*_w_, for the same reaction pair. Figure 9a shows that *M*_w,inst_ for the oxygenated reaction is well below the value for the reaction with no O_2_; *M*_w,inst_ goes as low as 2 × 10^5^ g/mol with O_2_, whereas at the same conversion, the reaction without O_2_, *M*_w,inst_, is 4 times greater, 8 × 10^5^ g/mol. These results, along with the same trend conveyed in Figure S5, indicate that O_2_ acts as a chain termination agent before the plateau; O_2_ lowers the chain length, just as a CTA does, but also terminates propagating radicals and slows propagation, which CTAs do not. Once the O_2_ flow stops *M*_w,inst_ increases as O_2_ is automatically eliminated on the conversion plateau until it reaches the same values as the no-O_2_ reaction, after all O_2_ has been eliminated.

Figure 9b shows RV_inst_ for the same reaction pair as 9a. The relationship of the two RV_inst_ curves very closely follows the behavior of *M*_w,inst_, shown in Figure 9a. This provides an important cross-check on the chain termination effect since the viscometer is a non-optical detector, independent of the light scattering data. This leads to a very interesting possibility—the use of O_2_ as a control variable to modify *M*_w_ during a reaction in a reversible manner, which is not possible with regular CTAs, such as sodium formate, which, once added into the reactor, cannot be easily removed.

**Figure 10:**
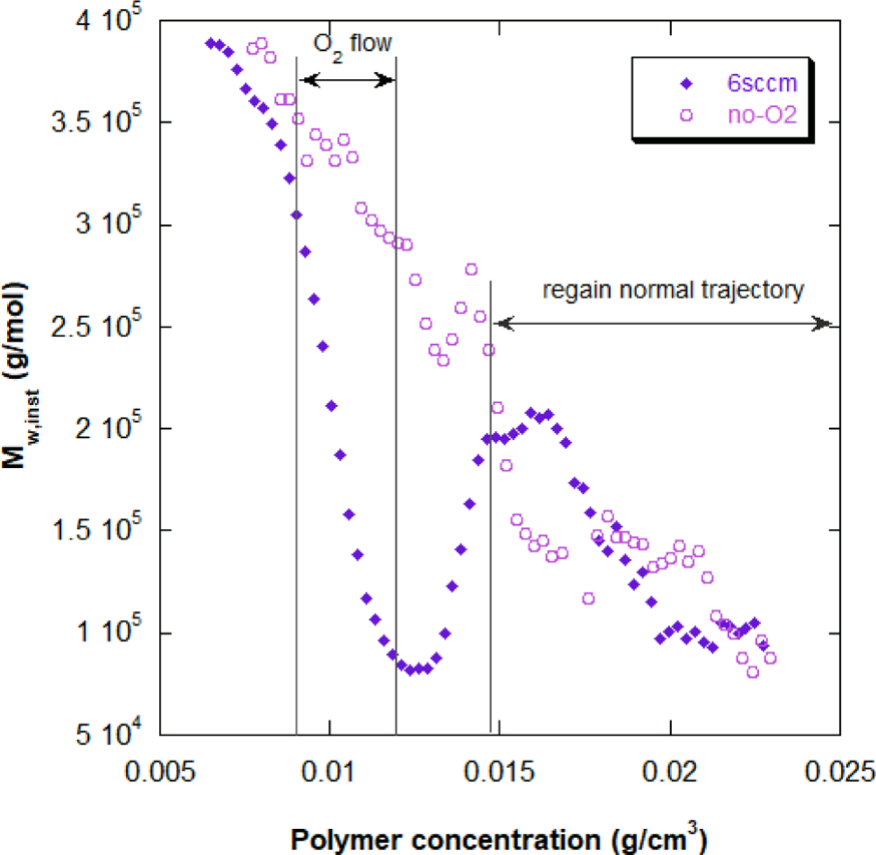
Instantaneous weight-average molecular weight (*M*_w,inst_) for RP of SS, case (ii), with 6 sccm airflow and with no O_2_ (reactions 1B and 2B in Table 2, respectively). The effect of O_2_ as a CTA decreasing *M*_w,inst_ is very large. Once the reaction resumes after O_2_ elimination by purging with N_2_, *M*_w,inst_ for 6 sccm rejoins the values for the reaction without O_2_.

In order to further assess the feasibility of an O_2_ flow rate as a control variable to obtain desired *M*_w_, the same procedure was tested using SS—with identical reaction conditions. Figure 10 shows the same chain termination effect on *M*_w,inst_ for RP of SS; *M*_w,inst_ with O_2_ is 4 times smaller at its minimum value than without O_2_; 0.75 × 10^5^ versus 3 × 10^5^ g/mol. It is important to point out, however, that in the SS case, O_2_ was not automatically eliminated, and another round of N_2_ purge was required for conversion to resume, thus forcing *M*_w,inst_ closer to that of the non-oxygenated reaction.

## 4 Kinetic Model

After thoroughly studying these three different cases and analyzing all the data of the different model systems and control experiments, a kinetic model can be proposed to account for all of the above observations.

### 4.1 Model for RP

The following model accounts for O_2_ and its inhibitory role: radicals from Am* and propagating pAm radicals, R* are transferred to O_2_ in case (i), and likewise for SS, in case (ii). In all cases, there is a threshold concentration of O_2_, [O_2_]_t_, at and above which this transfer of radical to O_2_ is so dominant that it stops the propagation reaction. Below [O_2_]_t_, the effect of O_2_ is to both slow the polymerization reaction and to terminate R*, producing shorter chains than when no O_2_ is present. The radical termination process by O_2_ slows the reaction and does not initiate any new chains. In contrast, a CTA does not slow the reaction but initiates new chains, as evidenced in work where multi-modal populations were obtained through a controlled feed of sodium formate.^31^

#### 4.1.1 Am as a Catalytic CTA

The data compel the following interpretation of events at the beginning of and during the conversion plateau: as [O_2_] increases due to airflow into the reactor, it reaches [O_2_]_t_, at which point chain propagation ceases and O_2_ now traps radicals only from Am* instead of pAm* (and likewise for SS* and pSS*), since polymerization ceases on the plateau. Interestingly, for RP of Am, case (i), this is a radical propagation cycle, not a termination event, because when O_2_ removes the radical from Am*, this latter returns to the state of an intact Am monomer and is not terminated, as is the case when O_2_ traps a radical from R* and produces a dead polymer chain. The radical O_2_^*^ goes on to react with some agent, most likely water, to produce a final “dead product with O_2_^′^”, DPO_2_, that removes the free O_2_ from the solution.

**Scheme 1:**
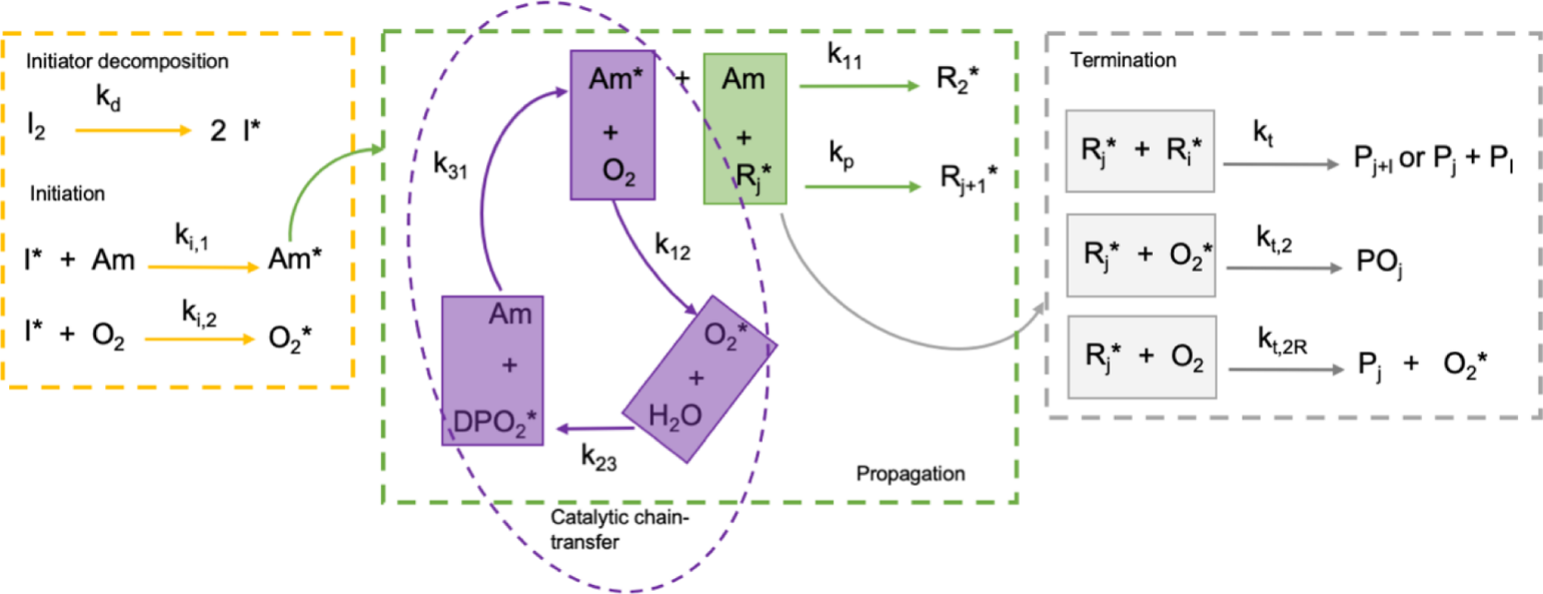
Block Representation of the Kinetic Scheme for RP of Am, Case (i), in the Presence of O_2_ The block visualization of the processes involved in the kinetic model includes the catalytic chain-transfer step (encircled in purple).

In this process, the radical is returned to Am to regenerate Am*, which then repeats the cycle. Hence, for case (i), what occurs on the conversion plateau is the elimination of free O_2_ from the solution through a cycle with Am* where Am acts as a catalytic radical-transfer agent. It is catalytic because it is not consumed, at least not detectably. The reason that the conversion appears to stop so abruptly is that the radical-transfer constant, *k*_12_, from Am* to O_2_ (and SS* to O_2_) is several orders of magnitude larger than the R* propagation constant *k*_p_, so that the effect of O_2_ on propagation is large and grows over a narrow range of very low [O_2_]. These effects will become apparent via the scheme and rate equations below. Scheme 1 summarizes this cyclic process for case (i), where O_2_ is eliminated by Am.

This process might be similar to the role of the enzyme glucose oxidase (GOx), which is effective at degassing reaction media at very low concentrations and through which O_2_ turns into H_2_O_2_,^7^ a compound that would not be identifiable using an RDO probe, for example. Another possibility is Am doing the combined work of GOx and sodium pyruvate, as proposed by Enciso and co-workers.^9^ Am could also be seen as a “fish” as it leads to the depletion of dissolved O_2_, just as fish do. However, mechanistically, Am is analogous to hemoglobin since it does not directly consume O_2_ itself but binds with it and then releases it at a catalytic site where O_2_ is finally consumed in a reaction. If the system was to be treated as a copolymerization, then the reactivity ratio of Am would be less than that of O_2_ since Am remains constant on the plateau, whereas O_2_ drops. In fact, the reactivity ratio of Am is zero if there is no conversion at all of Am.

#### 4.1.2 Rate Equations

Usually, Am* would simply be represented as R_1_^*^ the first initiated monomer, leading to chain propagation. Here, because Am* is regenerated when O_2_ is present, it has its own important mechanistic distinction from higher radicals R_2_^*^, R_3_^*^, and so forth and the corresponding rate equation. Hence, in the following, R_total_^*^ refers to all propagating radicals R_1_^*^, R_2_^*^, R_3_^*^, and so forth, whereas R* refers to R*_total_–R_1_^*^, that is, R* is R_2_^*^, R_3_^*^, and so forth.

In the kinetic model, “1” refers to Am, “2” to O_2_, and “3” to the product formed upon O_2_ elimination, DPO_2_.

The individual steps conveyed by Scheme 1 are given by the following reactions and associated rate constants:

1 Initiator decomposition

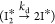

2 Am initiation, formation of the first propagating radical

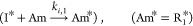

3 First Am propagation step

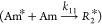
where *k*_*i*,1_ is the initiation rate constant and *k*_11_ is the rate constant for the first propagation step Am* + Am = R_2_^*^. Step 3 is explicitly singled out because it is the “gateway” step to propagation of R* > R_2_^*^ and is critical to the O_2_ threshold effect, as detailed below. If step (3) does not occur, then there is no subsequent chain propagation.

4 Radicalization of O_2_ by the initiator,

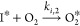
which also leads to O_2_ elimination. As demonstrated in Figure S3, however, the concentration of KPS, which generates the primary radicals I*, is too low in all of the Table 2 reactions to eliminate a measurable amount of O_2_.

5 Radical transfer from Am* to O_2_, the first step in O_2_ elimination

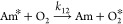

6 O_2_^*^ reacts with something else, likely water, eliminating O_2_

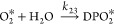

7 End of free O_2_ elimination cycle, formation of dead O_2_ product, return of radical to Am

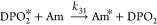


All the data indicate that the return of the radical to Am* is logically necessary, both to cause the observed threshold [O_***2***_***]***_***t***_***and to explain the lack of consumption of Am.***

8 O_2_^*^ terminates the propagating radical R_j_^*^, of degree of polymerization j, to dead polymer chain P_j_

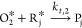

9 O_2_ terminates R_j_^*^ to dead polymer P_j_ and O_2_ becomes radicalized to O_2_^*^

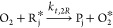

10Propagating radicals terminate each other by recombination or disproportionation

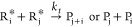

11 Main polymer propagation mechanism, *k*_p_, may vary with chain length

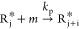


The reactions imply the following balance equations
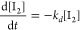
4
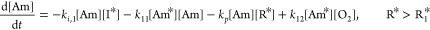
5

***Here, the positive term***, is the transfer of the radical from Am* to O_***2***_***, regenerating Am. Without this positive term, the threshold condition d[Am]/dt = 0 cannot be reached.***
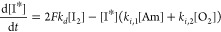
6
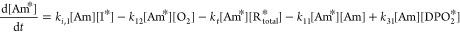
7
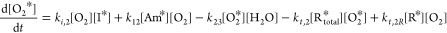
8
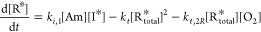
9
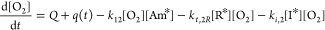
10where *Q* is the constant rate of O_2_ flow into the reactor from the compressed airflow and *q*(*t*) is the rate at which O_2_ backflows from the headspace into the reactor liquid. As long as O_2_ remains in the headspace, there will always remain a low level of O_2_ in the water due to the positive O_2_ source term from headspace to water. This will gradually taper off as the slowly decomposing initiator is used up and/or as the O_2_ in the headspace is very slowly depleted. Therefore, strategies that limit the headspace, making it extremely low, or zero, such as in ref (17), should allow an oxygenated Am reaction to proceed after O_2_ elimination. With a large headspace, it might still be possible with a steady initiator feed over a very long time.

The term  in eq 10 is responsible for the O_2_ consumption in Figure S3, which had a very high concentration of KPS ([KPS] = 0.185 mol/L) but no Am, and in which no polymerization reaction occurred. However, as also seen in Figure S3, for the experiment with low concentration KPS ([KPS] = 3.77 × 10^–3^ mol/L) and no Am, this term is negligible when modeling the kinetics of the polymerization reactions in Table 2.

The kinetic chain length, ν, is given by the probability of propagation divided by the probability of termination, either by R_total_^*^R_total_^*^ recombination/disproportionation or by termination via radical transfer to O_2_
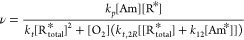
11

If O_2_ competes with Am for I*, then conceivably [R*] would be lower than in the absence of O_2_ and so ν could actually be larger than in the absence of O_2_, except that the data show that the effect of *k*_*t,2R*_ is much greater than the reduction of R* in the denominator, and so the kinetic chain length is shortened by O_2_, not increased by a reduction in [R*].

##### 4.1.2.1 Origin of the Oxygen Threshold, [O_2_]_t_, for Stopping the RP

***The experimental observation is that there is a threshold level of O***_***2***_***, [O***_***2***_***]***_***t***_***, at which Am ceases to be consumed; that is, for [O***_***2***_***] ≥ [O***_***2***_***]***_***t***_***,******eq 5******becomes d[Am]/dt = 0. This can occur only because of the positive term,******, in******eq 5***, due to Am* transferring the radical to O_***2***_***and regenerating Am.*** This leads to the threshold condition
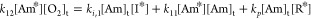
12where [Am]_t_ is the concentration of Am at the threshold. It requires the rate constants and radical concentrations to solve for the threshold condition. These are not readily available. However, as the threshold is approached, two simplifying approximations can be made. First, since propagation for R* > R_1_^*^ effectively ceases at the threshold—the ACOMP data show no conversion or chain production on the plateau—the term *k*_*p*_[Am][R*] can be ignored in eq 12. Similarly, a relatively small amount of Am* is produced by direct initiation since KPS is in such low concentration and initiator decomposition is slow, so that *k*_*i*,1_[Am]_t_[I*] ≪ *k*_11_[Am*][Am]_t_ in eq 12. This point is addressed further in Section 4.4*.* This leads to the approximation
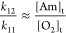
13where [O_2_]_t_ is the measured threshold value of [O_2_] upon the abrupt turn-off (or re-start) of the reaction and [Am]_t_ is the measured value of [Am] when the reaction turn-off occurs (which is equal to [Am] when the reaction re-starts since there is no Am consumption on the plateau).

Because of mixing issues and the low end of resolution of the O_2_ probe, it was difficult to obtain accurate and consistent measurements of [O_2_]_t_. However, current data suggest that the threshold concentration is [O_2_]_t_ = 0.58 × 10^–6^ g/cm^3^, *or lower*, so [O_2_]_t_ = 1.66 × 10^–8^ mol/cm^3^. The Am level for this from ACOMP data is 0.022 g/cm^3^ = 3.09 × 10^–4^ mol/cm^3^. This gives a *lower limit* for the ratio
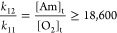


This shows that the rate constant for transfer of radical from Am* to O_2_ is *at least* 18,600× greater than for Am* adding a monomer Am. At this [O_2_]_t_, the kinetic chain length in eq 11 will be vanishingly small, another indication that polymerization has stopped on the conversion plateau.

Following the notion that O_2_ elimination proceeds chiefly by radical transfer from Am* to O_2_ and using *Q* = *q*(*t*) = 0 in eq 10 allows it to be approximated by
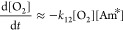
14

Then, using the approximate forms of eqs 5 and 10 gives
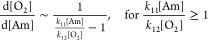
15

This ratio diverges when d[Am] = 0, and there is no Am consumption as was experimentally observed for all conversion plateaus.

**Figure 11:**
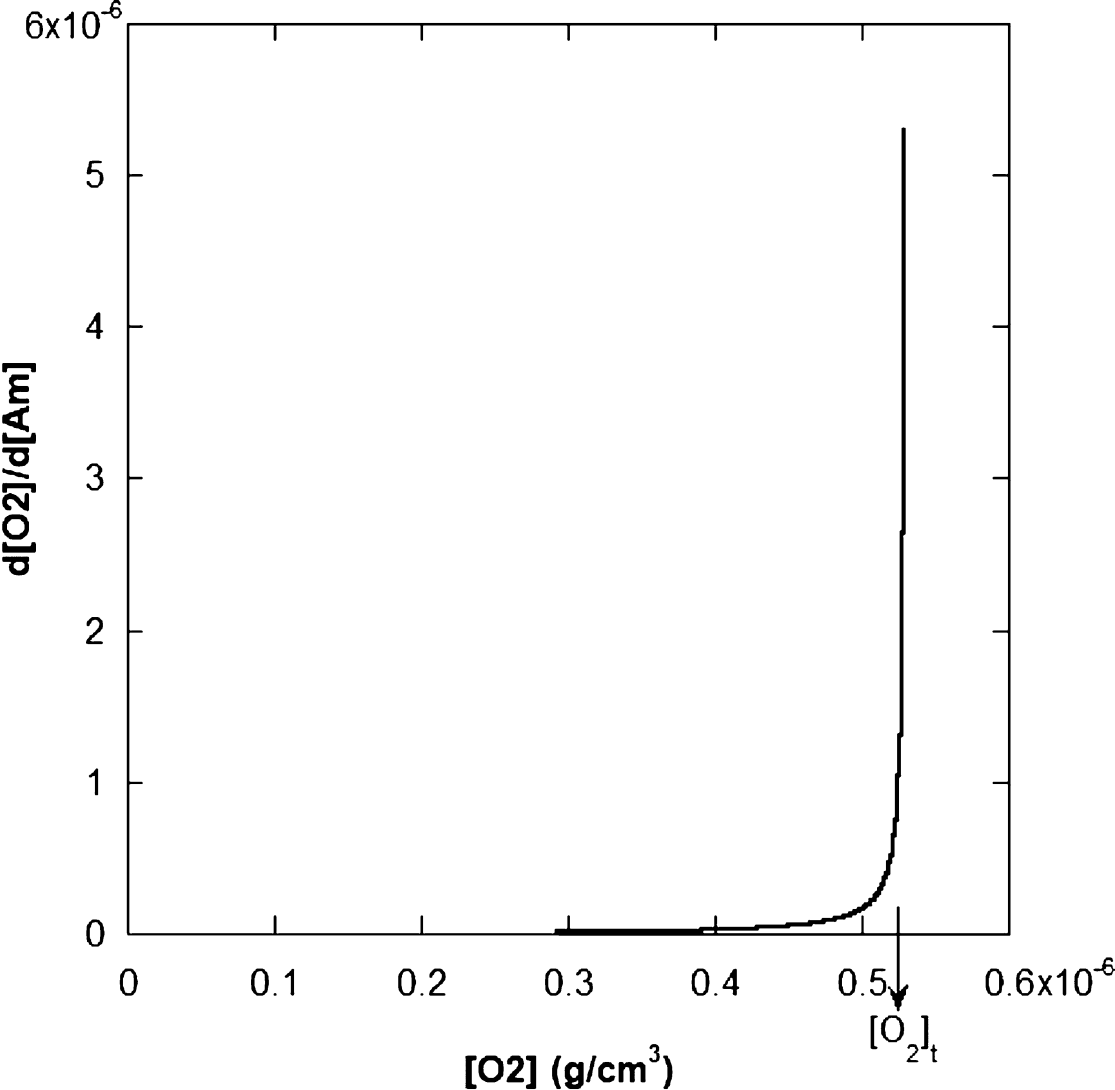
Threshold [O_2_]_t_ for turn-off and re-start of the Am polymerization reaction 8A in Table 2 using the approximate eq 15.

Figure 11 shows schematically how eq 15 diverges as [O_2_] increases, producing the observed threshold for reaction turn-off and re-start. Figure 11 uses the lower limit of *k*_12_/*k*_11_ = 18,600 to illustrate the effect. At the start of the reaction, while O_2_ is flowing into the reactor, [O_2_] increases until [O_2_]_t_ is reached, and the reaction turns off. When sitting on the conversion plateau, [O_2_] is decreasing but, still, [O_2_] > [O_2_]_t_. As [O_2_] continues to decrease, according to the above model, the threshold is again reached, and the reaction re-starts.

### 4.2 Case of SS RP (Case (ii))

Recalling the data in Figure 5 for SS, the same type of reaction turn-off threshold and conversion plateau were found, but the reaction did not spontaneously re-start; that is, SS does not eliminate O_2_ at a rapid rate, whereas Am does. The threshold condition in eq 13 requires that SS* transfers its radical to O_2_ and restores SS, as in eq 5, with the positive term , where [SS*] and [SS] replace [Am*] and [Am] in eq 5. For this type of threshold behavior, and substituting Am for SS, eq 13 becomes
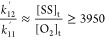
where *k*_12_^′^ is the rate constant for transfer from SS* to O_2_ and *k*_11_^′^ is the rate constant for the first propagation step SS* + SS = R_2_^*^. [O_2_]_t_ was difficult to determine, but considering the second flow rate period in Figure 5, it was reasonable to use the upper value of 0.58 × 10^–6^ g/cm^3^ from the Am reactions, thus furnishing the lower limit of 3950. Given the uncertainties in [O_2_]_t_, it is not possible to say if the differences in lower limits for *k*_12_/*k*_11_ and *k*_12_^′^/*k*_11_^′^, between Am and SS, respectively, are significant. Both show, at any rate, that the radical-transfer constant from monomer to O_2_ is very large compared to the rate constant for the first propagation step. There could be differences due to the much smaller propagation rate constant of SS compared to Am (on the order of 10^2^ lower) and to the lower energy and greater stability of the radical SS* compared to Am* due to the conjugated styrenic ring.^35,36^

The radical Am* has much higher energy than both O_2_^*^ and SS* and can transfer its free radical to whichever is present. In fact, it was recently found that the presence of even small amounts of SS, when copolymerized with Am, leads to a deceleration of the Am conversion because Am* tends to transfer its radical more readily to SS than to propagate via addition of Am.^33^ The fact that O_2_ completely stops Am polymerization, whereas SS merely slows Am polymerization, suggests that the energy of O_2_^*^ is lower than that of SS*. This could lower *k*_12_^′^ for SS* radical transfer to O_2_ enough that O_2_ is only slowly eliminated by SS on the conversion plateau.

### 4.3 Model for RAFT (Case (iii))

The major difference in O_2_ effects for RP and RAFT for Am is that O_2_ is eliminated on the plateau for RP, case (i), leading to a spontaneous re-start, but that O_2_ is not eliminated on the plateau for SS RP, case (ii), nor for Am RAFT case (iii). ***Before explaining this, it is important to point out that the lack of O***_***2***_***elimination in RAFT is not due to the fact that [KPS] in RAFT was 5× to 10× less concentrated than in the RP experiments because the higher [KPS] in the RP reactions had no role in the measured O***_***2***_***elimination process.***

The following is a hypothesis to explain the lack of O_2_ elimination in RAFT. In the equilibrium phase of the RAFT process, propagating radicals dock reversibly to the RAFT agent, add Am monomers when they dissociate from the RAFT agent, and then re-dock and go dormant, until again dissociating, in a reversible docking and dissociating cycle, or “reversible addition and fragmentation chain transfer”. A major difference with RP is that in RAFT, Am is added to active radicals, pAm*, when they dissociate from the RAFT agent. The concentration of pAm* is determined by the initial ratio of monomer to RAFT agent, given that this quantity controls the number of chains, based on the target *M*_w_. Am* produced directly by the initiator (where [KPS] is around an order of magnitude lower than in the RPs) is at a very low concentration and so has no detectable activity as a catalytic radical-transfer agent that eliminates O_2_. It is also unknown whether radicals R_2_^*^ and higher have the same radical-transfer rate to O_2_ as Am*. The hypothesized reason that O_2_ shuts down the RAFT process is that O_2_ also docks reversibly with the RAFT agent, but with a higher binding affinity than the active pAm* radicals. Since the O_2_ docking is reversible, there is no destruction of either O_2_ or RAFT agent. Furthermore, according to Moad and co-workers,^6^ the O_2_ molecules preferentially bind to the intermediate adducts formed during the RAFT mechanism; therefore, once that happens, they end up blocking the fragmentation of the growing polymer chains and essentially cause the RAFT polymerization to simply “freeze”. This is consistent with what is shown in Figure 7 and could be somewhat compared to the slow fragmentation hypothesis, which is ubiquitous for all RAFT polymerization regardless of the CTA type.^37^ More specifically, rate retardation when using dithiobenzoate agents for acrylic and styrenic derived monomers has been extensively studied, showing that trithiocarbonate agents—like the one used here for Am and SS—are better suitable.^38,39^ However, here we see more than just retardation and the actual halting of the polymerization process while O_2_ levels are above the threshold. Once O_2_ is purged by N_2_, the RAFT process continues as no O_2_ had ever been injected into the reactor medium, and control is regained.

It is important to point out that although the RAFT agent intermediate radicals are sensitive to O_2_, what is found here is that they are not irreversibly deactivated when attaching to/in the presence of O_2_, instead of growing polymer chains. RAFT agent oxidation has been investigated for a variety of CTA molecules with aromatic moieties in organic solvents, showing that thiol portions turn into carbonyl portions.^40^ This would obviously affect the RAFT process in a complex manner as it changes the affinity of the different radicals to the RAFT molecule itself. However, none of the RAFT agents explored in reference (40) resembles the BM1433 used here, so, combined with all experimental evidence, oxidation of the CTA molecule itself seems unlikely.

In case (iii), because of the competition between Am (holder of the radical at the end of the propagating pAm* radicals) and O_2_ for binding to the RAFT agent, only a fraction F of RAFT sites is available to the docking/dissociating propagating radicals pAm*, controlled by the respective binding constants *b*_Am_ and *b*_O_2__ for Am and O_2_. This makes the Am consumption rate proportional to *F*[Am]
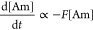
16where *F*, up to RAFT agent saturation by O_2_, is given by
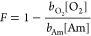
17

Am conversion stops when *F* = 0; that is, the threshold occurs when
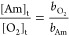
18where [Am]_t_ is the concentration of Am upon reaction turn-off. The fact that at the threshold [Am] ≫ [O_2_] implies that *b*_O_2__ ≫ *b*_Am._. Equation 18 has the same form as eq 13, with *b*_O_2__ and *b*_Am_ replacing the radical-transfer constants *k*_12_ and *k*_11_, respectively. By visual inspection of data for reaction 1C (see Figure 6), a [O_2_]_t_ of 0.2 × 10^–6^ g/cm^3^ can be estimated. It was found that [Am]_plateau_ = 0.022 g/cm^3^, so that
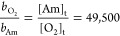


This means that the binding constant to O_2_ is around 50,000× greater than that of the Am moiety that holds the radical on active pAm* chains, which will inevitably stop polymerization while O_2_ is present in the reaction medium, causing the observed abrupt shut-off among all the three cases. Again, the value of [O_2_]_t_ ∼ 0.2 × 10^–6^ g/cm^3^, is only approximate, due to the mixing and O_2_ probe resolution issues mentioned earlier. However, this shows that the binding of O_2_ to the RAFT agent is on the order of 10^4^ or higher, just as radical transfer from both Am* and SS* to O_2_ was on the order of 10^4^ or higher than propagation. Since CTA molecules with a trithiocarbonate group (as BM1433) produce less stable intermediate adducts,^6^ this estimated O_2_ binding constant, *b*O_2_, could be even higher if other more oxygen-sensitive CTA types are employed.

Another possible explanation, even though rather unlikely, is that O_2_ actually “steals” the radical from the intermediate RAFT molecule adduct. In either case, the proposed Am catalytic cyclic mechanism for O_2_ consumption may also be happening, but at a much lower rate, given the lower [KPS]. However, no full explanation can be given solely using the data presented here, so more experiments would be required to test these multiple hypotheses.

Hence, although both RP and RAFT show the sharp turn-off threshold, the proposed mechanism causing it is actually different in each. It is important to point out that the rate of approach to the turn-off threshold for both RP and RAFT reactions increases with the compressed airflow used, although the existence of the turn-off threshold is independent of the airflow rate. Once there is no active introduction of O_2_ into solution, that rate is governed by the backflow from headspace and the Am cyclic catalytic O_2_ consumption, without ever affecting the [O_2_]_t_ itself. Thus, [O_2_]_t_ is completely independent of O_2_ source (compressed air or backflow from the headspace) but is most likely dependent on the solvent, a variable that has not been explored here but could lead to interesting follow-up work.

### 4.4 Estimation of Am Catalytic Efficiency for Case (i)

A detailed analysis of the catalytic efficiency of Am and the estimate of the amount of O_2_ elimination is given in Section S3 of the Supporting Information. This analysis takes into account flow rates, headspace to reactor O_2_ backflow, long-term O_2_ equilibrium under flow, the kinetic equations mentioned above, and several other factors.

Aside from the detailed analysis in the Supporting Information, another way of quickly estimating the catalytic efficiency in case (i) is to consider the number of free radicals generated over 16,000 s from KPS decay. At 50 °C, the decomposition rate for KPS is *k*_D_ = 1.7 × 10^–6^ s^–1^. With an initial [KPS]_0_ = 1.6 × 10^–6^ mol/cm^3^, the number of free radicals generated per cm^3^ (two I* generated per KPS decomposition) is
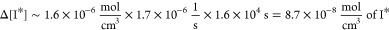


Since the amount of O_2_ eliminated over 16,000 s is ∼1.14 × 10^–6^ mol/cm^3^, a single I* must suffice to eliminate at least 1.14 × 10^–6^/8.7 × 10^–8^ = 13 molecules of O_2_. Since the elimination is mediated by Am (no elimination occurs in the absence of Am at this low initiator concentration), ***each Am must be able to go through at least 13 catalytic cycles, and likely more, before being used up in a side reaction (e.g., termination).***

## 5 Summary

The effect of O_2_ concentration on Am polymerization creates a threshold phenomenon for both RP and RAFT, cases (i)–(iii), occurring at a certain critical concentration of O_2_, [O_2_]_t_, which depends on reaction conditions. At [O_2_]_t_, the conversion of a monomer to a polymer abruptly stops and a flat plateau of zero conversion versus time occurs, for all cases, on which there is no further detectable polymerization, and no further loss of monomer by any mechanism, as long as O_2_ remains at or above [O_2_]_t_. For case (i), the O_2_ during the plateau is eliminated spontaneously and fairly rapidly via a postulated rapid Am radical-transfer catalytic cycle, and once it drops to [O_2_]_t_, conversion resumes as if O_2_ was never present, approaching non-oxygenated values for reaction rate, RV_inst_, and *M*_w,inst_. During the plateau of case (i), Am acts as a radical-transfer catalyst in the process that eliminates O_2_, while O_2_ acts as a highly efficient inhibitor against Am polymerization. Am is catalytic in case (i) because it both dramatically facilitates the elimination of O_2_ and is not measurably consumed in the process. It is emphasized that the concentration of KPS during the reactions in Table 2 was too low to cause any detectable O_2_ elimination due to primary radicals from KPS decomposition.

For RP, cases (i) and (ii), while [O_2_] < [O_2_]_t_, O_2_ acts as a reversible, chain termination agent, shortening pAm and pSS chains, and slowing down the reaction. It is a reversible agent in the sense that once O_2_ inflow stops, O_2_ is eliminated naturally in case (i) and can be quickly purged away by N_2_ in all cases. Once below [O_2_]_t_, the reaction resumes and continues without further effects from the previous presence of O_2_. Since O_2_ can also be eliminated by direct N_2_ purge in all cases, fine control of O_2_ inflow might be a useful way of controlling the trajectory of *M*_w_ during RP reactions. This is not to be taken as a generalized statement since for other non-aqueous systems, it would require scrutinizing O_2_ solubility in their specific conditions.

In summary, O_2_ can have two major effects on polymerization reactions. First, in all cases, the polymerization reaction abruptly stops at the threshold [O_2_]_t_. The above kinetic model requires that the monomer radical, both Am* and SS*, transfers its radical to O_2_ with much greater probability than adding a monomer and propagating in cases (i) and (ii). While technically it was difficult to get an accurate measurement of [O_2_]_t_, due to mixing issues and sensitivity of the O_2_ probe, the transfer constant from monomer radical M* to O_2_, *k*_12_, is at least 4 orders of magnitude greater than the initial polymer chain propagation step, M* + M = R_2_^*^, with constant *k*_11_. For RAFT, case (iii), O_2_ can reversibly bind to the RAFT agent, thus shutting down the addition fragmentation chain-transfer propagation process, also in a predicted threshold process. Second, in case (i), the O_2_ above [O_2_]_t_ is eliminated by a catalytic cycle involving Am* radical transfer, and the reaction spontaneously resumes. No loss of Am was detected during O_2_ elimination in case (i), and it was estimated that Am goes through at least 13 catalytic cycles of O_2_ elimination, and likely much more, before being lost through a side reaction, such as termination. This effect does not occur for cases (ii) and (iii), although O_2_ elimination by SS is suspected to occur, but at a much lower rate than for Am, and was not experimentally measurable.

For cases (i) and (ii), it is surmised that it is the very low energy state of the radical O_2_^*^ that allows preferential radical transfer from monomer M* to O_2_, instead of propagation of M* into a polymer chain. For RAFT, case (iii), O_2_ can most likely bind strongly but reversibly to the RAFT agent, shutting down chain propagation. The energy order of radicals is Am* ≫ SS* > O_2_^*^. SS* obtains its low energy through the resonance of the styrene ring. It is not clear why the radical on the putative product DPO_2_^*^ is recycled back to Am quickly enough to cause spontaneous O_2_ elimination, case (i), whereas, if there is any recycling of radical from DPO_2_^*^ back to SS, case (ii), the process is not rapid enough to cause O_2_ elimination on the time scale of the reactions, nor dominate over O_2_ backflow from the headspace.

This work is intended to elucidate the O_2_ effects in polymerization reactions and fuel the discussion and modeling regarding its role in both RP and RAFT. It also sets the stage for experimental investigation of other polymerization systems with different solvents, monomers, initiators, concentrations, and reactor geometries. Further continuation of this work could focus on exploiting controlled O_2_ flow rates to tune copolymer composition, in addition to the molecular weight trajectory for RP, as has been successfully done in this group through the monomer semibatch approach.

## Supporting Information Available

Other detailed oxygenated experimental conditions for all experiments (reaction and control); initial conditions for control experiments; more detailed methods for reaction monitoring with ACOMP and GPC analyses; expanded discussion of O_2_ in water versus headspace; O_2_ solubility parameters; water versus headspace control experiments at different liquid volumes; control experiments with the initiator alone; reaction in situ monitoring of O_2_ concentration in water versus in the headspace; cumulative *M*_w_ over time for oxygenated and non-oxygenated reactions; and detailed discussion of Am catalytic efficiency for case (i). (PDF)
