# Supplementary material for: Observation and Modeling of a Sharp Oxygen Threshold in Aqueous Free Radical and RAFT Polymerization
Source: J Phys Chem B. 2022 Dec 15;126(51):10933–47. doi: 10.1021/acs.jpcb.2c06067 (PMC9806832; doi:10.1021/acs.jpcb.2c06067)
Supplement: Supplementary file 1 — jp2c06067_si_001.pdf [file jp2c06067_si_001.pdf]

SUPPORTING INFORMATION

Observation and Modeling of a Sharp Oxygen Threshold in Aqueous Free Radical and RAFT Polymerization

Julia S. Siqueira[1*], Matthew Crosley[2], Wayne F. Reed[1]
[1]Tulane University, New Orleans, Louisiana, 70118, USA
[2]Fluence Analytics, Stafford, Texas, 77477, USA

[*]Corresponding author email: jsiqueir@tulane.edu

Table of Contents

S1. Detailed experimental conditions for all experiments……………………………..……..S2

S1.1. Experimental Conditions ……………………………………………………..…S2

Table S1. List of reaction detailed oxygenation and plateau conditions ……..…S2

Table S2. List of control experiments with initial conditions ………....……….S2

Table S3. List of control experiments detailed oxygenation and plateau conditions ……………………………………………………………………………… ……..S3

S1.2. Reaction Monitoring …………………………………………………………..…....S4

Figure S1. Experimental setup employed showing monitoring by online technique ACOMP and in situ RDO probe. Also shown are some of the parameters automatically calculated on the ACOMP proprietary software. PE = extraction pump, GFM = gas flow meter/controller, T = stir + heat plate temperature probe. Details on the dilution stages used on the ACOMP system were omitted for simplification…………………………………………………………..……S4

S1.3. Offline GPC Analyses ……..……………………………...………………………..S4

S2. Oxygen in solution versus headspace and its effects during RP…………………..…....S4

S2.1. O_2 in water ……………………….……………………………..………………S4

Table S4. Summary of O_2 solubility and initiator dissociation parameters ......…S4

Figure S2. O_2 vs. time for solution and headspace experiments at different volumes. All experiments were carried out with compressed air flow at 75 sccm – 500 ml/100 ml water/headspace monitored in (1) headspace and (2) solution and 250 ml/350 ml water/headspace monitored in (3) headspace and (4) solution. The concentration in the headspace is relative, but α can be accurately determined. Values of α_{fit} and $\alpha_{Eq.2}$ in the table were obtained following Equation 1 and Equation 2, respectively. ………………………………………………….……S5

Figure S3. Control experiments showing O_2 concentration in solution for KPS in water at different concentrations – 1.85×10^{-1} mol/L (dashed black line) and 3.78×10^{-3} mol/L (continuous gray line)…..S6

S2.2. Reaction O_2 Monitoring ………………………………………………..………..S6

Figure S4. Solution (blue line) and headspace (black line) O_2 concentration monitoring during Am RP reactions carried out under the same conditions (reactions 9A and 10A in Table 1, respectively). The diamonds in each curve mark the beginning and the end of compressed air flow period. The concentration in the headspace is relative, but α can be accurately determined. ……………………………………………………………………………...S7

Figure S5. Cumulative weight average molecular weight (Mw) for polymerization of Am with 6 sccm airflow and with no O2 (reactions 12A and 13A in Table 1, respectively). S8
S3. Full estimation of Am catalytic efficiency for case (i)…………………………………S7

S1. Detailed experimental conditions for all experiments

S1.1. Experimental Conditions

Table S1. List of reaction detailed oxygenation and plateau conditions

Reaction (case)	O_2 flow duration (s)	Start flow rate to plateau (s)	Time on plateau (s)	$[O_2]$ at end flow rate (g/cm^3) x10^6	$[O_2]$ at peak (g/cm^3) x10^6	Peak to re-start (s)
1A (i)	No-O_2	-	-	-	-	-
2A (i)	4000	3983	428	-[a]	-[a]	422
3A (i)	6889	416	6861	-[a]	-[a]	388
4A (i)	3417	169	3714	-[a]	-[a]	466
5A (i)	778	186	858	-[a]	-[a]	266
6A (i)	No-O_2	-	-	-	-	-
7A (i)	3264	385	4064	4.58	4.74	3257
8A (i)	1758	259	3253	3.58	3.72	1744
9A (i)	1843	154	3244	3.56	3.67	1562
10A (i)	1862	503	2322	8.62	8.62	963
11A (i)	14609	1523	13146	5.50	5.56	-
12A (i)	2286	1208	1333	0.19	0.19	568[b]
13A (i)	No-O_2	-	-	-	-	-
1B (ii)	459	459	5037	0.27	3.36	1424[b]
2B (ii)	575	370	1053	0.26	0.49	500[b]
1C (iii)	1119	2866	427	0.01	0.01	310[b]
2C (iii)	No-O_2	-	-	-	-	-

[a]Not measured – RDO probe was not used due to its temperature limitations.
[b]No spontaneous re-start was observed, but reaction resume after O_2 purge with N_2.

Table S2. List of control experiments with initial conditions

Experiment	Figure	O_2 flow rate (sccm)	[Am] (mol/L)	[KPS] (mol/L)	[PAm] (mol/L)
(1)	3a	200	-	-	-
(2)		200	-	3.77x10^{-3}	-
(3)		200	0.478	-	-
(4)		Reaction 7A in Table 1			
(5)		200	9.56 x10^{-3}	-	0.468
(6)		15	-	-	-
Grey	3b	200	-	3.77x10^{-3}	-

Black	200	-	0.185	-

Table S3. List of control experiments with detailed oxygenation and plateau conditions

Experiment	Figure	O_2 flow duration (s)	Start flow rate to $[O_2]_{sat}$	$[O_2]$ at end flow rate (g/cm^3) x10^6	$[O_2]$ at peak (g/cm^3) x10^6
(1)		2197	2191	6.57	6.70
(2)		2184	2184	6.48	6.59
(3)	3a	2268	2268	6.34	6.58
(4)		Reaction 7A in Table SI1			
(5)		3735	1677	5.86	5.86
(6)		150	-	0.17	3.98
Grey	3b	3277	2139	6.10	6.10
Black		3194	2444	5.83	5.94

S1.2. Reaction Monitoring

ACOMP concentrations of Am and SS were calculated using the 250 and 260 nm raw UV signals, respectively. The reactor content stream was conditioned through two consecutive dilution stages, providing dilutions ranging from 20 to 81x, for the Am RP reactions, and from 10 to 18x for Am RAFT reactions. Given the strong UV absorption of the SS molecule, for the SS RP reactions, the dilution of the content stream going into the UV was increased substantially, to over 400x. Overall, total solids content (monomer + polymer) going through the UV ranged between 0.065×10^{-3} and 2.76×10^{-3} g/cm^3, while staying between 1.05×10^{-3} and 4.85×10^{-3} g/cm^3, for the other two detectors during RP and RAFT, respectively. Temperature and stirring were appropriately controlled, using a magnetic stirrer at the highest possible speed (RPM) setting – 1000 RPM – to ensure better mixing of the O_2 in the reactor content. Figure SI1 below provides a simplified illustration of the set up used for all reactions, including both ACOMP and RDO probe monitoring.

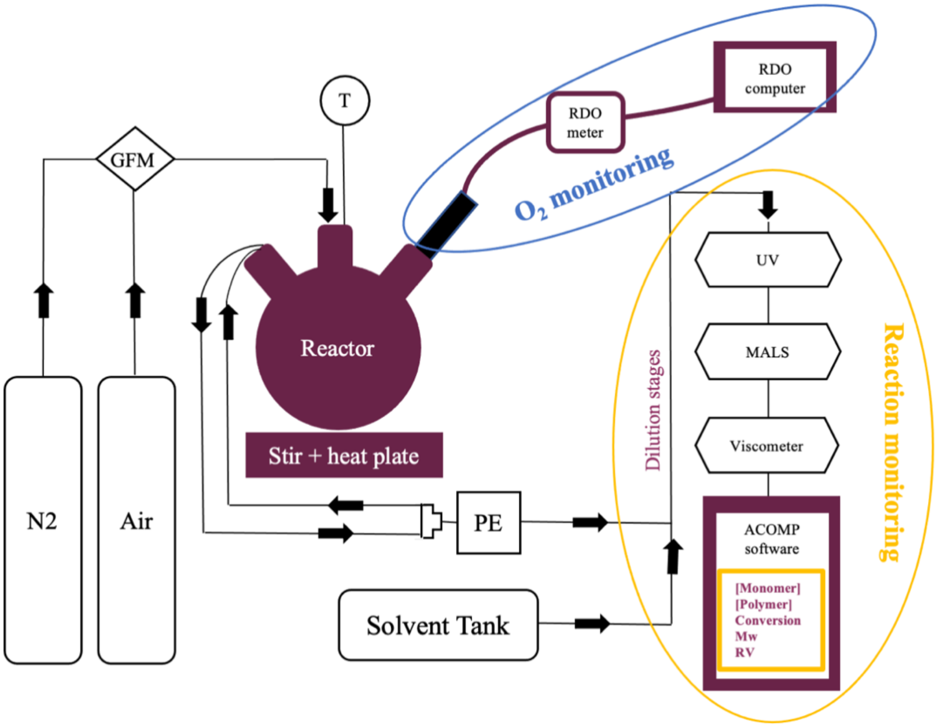

Figure S1. Experimental setup employed showing monitoring by online technique ACOMP and in situ RDO probe. Also shown are some of the parameters automatically calculated on the ACOMP proprietary software. PE = extraction pump, GFM = gas flow meter/controller, T = stir + heat plate temperature probe. Details on the dilution stages used on the ACOMP system were omitted for simplification.

S1.3. Offline GPC Analyses

Deionized water was used as the mobile phase for the GPC system, which consisted of a Rheodyne 7725i manual injector, with a 100μL sample loop, connected to a Shimadzu LC-10AT VP pump, and a Shimadzu SPD-10AV UV detector with a 1 cm flow cell, followed by a Shimadzu RID-10A refractive index detector. A Shodex OHpak SB-802.5 HW column was chosen for all analyses of the reaction aliquots, as it is able to separate monomer and low molar mass polymers, allowing to track any intermediate shorter polymer chains formed, which further confirms that no polymerization happened during the conversion plateau. The GPC data were custom analyzed.

S2. Oxygen in solution versus headspace and its effects during RP

S2.1. O_2 in water

Table S4. Summary of O_2 solubility and initiator dissociation parameters

Compound	Parameter	
Initiator (KPS)	Dissociation constant k_d @ 50 °C (1/s)	$1.70\text{x}10^{-6}$
	Dissociation constant k_d @ 65 °C (1/s)	$1.5\text{x}10^{-5}$

	d[I*]/dt @ 50 °C, t=0 (mol/cm^3-s)	5.64x10^{-12}
Air	Density @ STP[a] (mol/cm^3)	4.46x10^{-5}
Oxygen (O_2)	Density @ STP[a] (mol/cm^3)	9.37x10^{-6}
	Mass saturation in H_2O (g/cm^3) @ 50 °C	5.20x10^{-6}
	Molar saturation in H_2O (mol/cm^3) @ 50 °C	1.625x10^{-7}
	Mass saturation in H_2O (g/cm^3) @ 65 °C	4.5x10^{-6}
	Molar saturation in H_2O (mol/cm^3) @ 65 °C	1.41x10^{-7}
	Fraction dissolved in H_20 @ 50 °C	0.017
	Fraction dissolved in H_20 @ 65 °C	0.015

[a] Standard Temperature & Pressure (@ 273K, 1 atm)

To test Equations 1 and 2 (main manuscript), two analogous set of experiments with a compressed air flow rate of 75 sccm were carried out – one with 350 ml/250 ml headspace/water and one with 100 ml/500 ml headspace/water volumes (Figure SI2). For each of them, the O_2 concentration was monitored for both solution and headspace. The expected rise rate α was calculated through the exponential fitting of the [O_2] curves following Equation 1. The alphas calculated based on Equation 2 ($\alpha_{Eq.2}$) are also presented on the table in Figure SI2. As it can be seen when comparing the alphas obtained through the two methods, they are very close for the headspace experiments (1 and 3). However, the values predicted by Equation 2 for the solution experiments (2 and 4) are much higher than what's actually obtained according to Equation 1 exponential fit; the latter are actually very close together, indicating that in the solution case, O_2 rises as mostly exclusively dependent on the flowrate used and very little on the solution volume. The slower rise rates in solution is presumed to be limited by the dissolution of O_2 into the reactor liquid. It is important to point out that the [O_2] is only accurately measured in solution, which makes the headspace data magnitude data qualitative, but the rise rates are correct, once they stem from the exponential fit according to Equation 1.

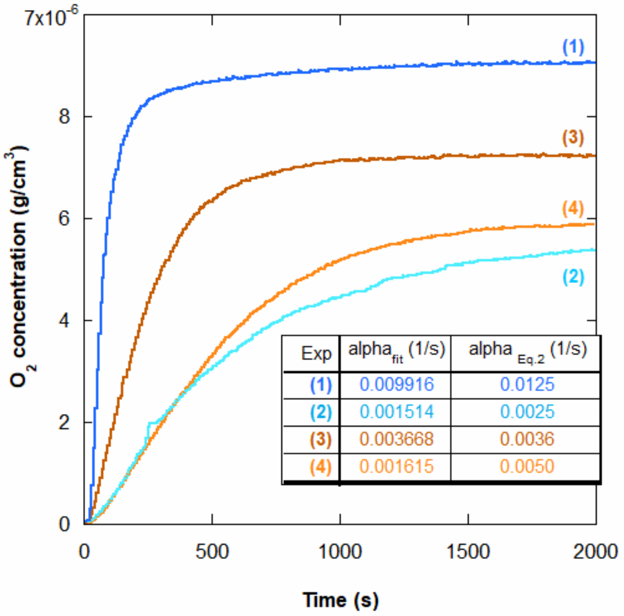

Figure S2. O_2 vs. time for solution and headspace experiments at different volumes. All experiments were carried out with compressed air flow at 75 sccm – 500 ml/100 ml

water/headspace monitored in (1) headspace and (2) solution and 250 ml/350 ml water/headspace monitored in (3) headspace and (4) solution. The concentration in the headspace is relative, but α can be accurately determined. Values of α_{fit} and $\alpha_{Eq.2}$ in the table were obtained following Equation 1 and Equation 2, respectively.

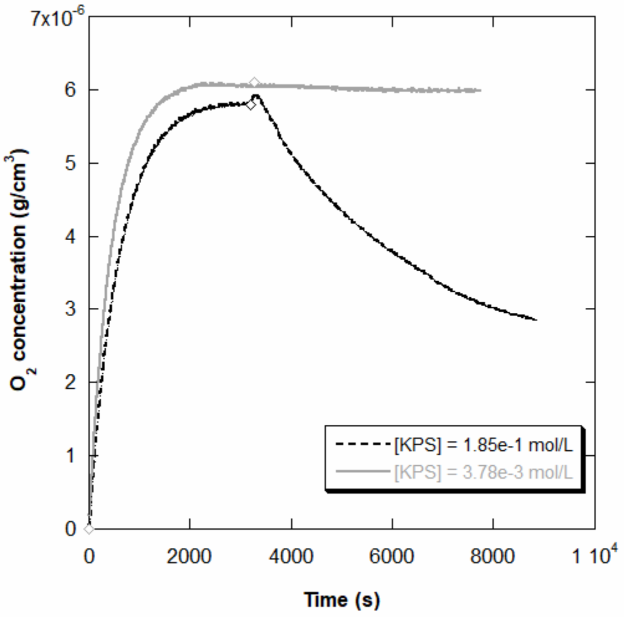

Figure S3. Control experiments showing O_2 concentration in solution for KPS in water at different concentrations – 1.85×10^{-1} mol/L (dashed black line) and 3.78×10^{-3} mol/L (continuous gray line).

S2.1. Reaction O_2 Monitoring

Figure SI4 shows the differences in rate between O_2 concentration in the solution and in headspace during RP reactions carried out under the same conditions - identical compressed air flow periods (1800 s, or 30 min) at 200 sccm, reactions 9A and 10A in Table 1, respectively). Although the headspace concentration data are just qualitative – O_2 concentration measurements are accurate only in solution – the O_2 rise value, α in Equation 1 (main manuscript), can be accurately determined and is very much different and, in accordance with Table 2 (main manuscript), the amount of O_2 in the headspace is significantly higher than in solution. Although the calculated value for $[O_2]_{plateau}$ in solution is 50% higher than that of the headspace, the fit only reaches the plateau after 50000 s, which is much longer than the 5000 s it would take to reach the observed values around 6 mg/L, from the control experiments. The same trend can be seen during the initial O_2 purge with N_2 at 75 sccm (time < 0) – $[O_2]$ in headspace drops dramatically right away since the headspace fills up quicker with N_2, as opposed to a much smoother reduction in solution. The table in Figure SI4 shows the calculated values from the 1st order fitting of the data for α_{fall} and α_{rise} – $[O_2]$ decrease rate during purge with N_2 and increase rate during compressed air flow period, respectively. Again, the values obtained for the headspace are at least one order of magnitude higher than those for the solution monitoring.

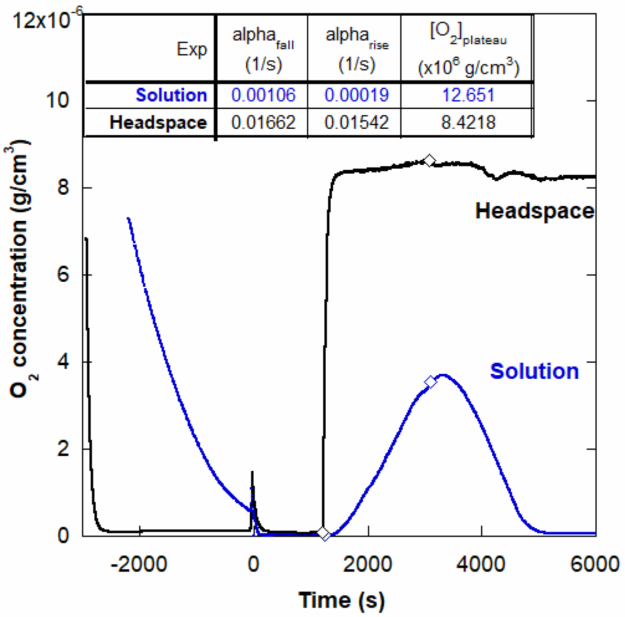

Figure S4. Solution (blue line) and headspace (black line) O_2 concentration monitoring during Am RP reactions carried out under the same conditions (reactions 9A and 10A in Table 1, respectively). The diamonds in each curve mark the beginning and the end of compressed air flow period. The concentration in the headspace is relative, but α can be accurately determined.

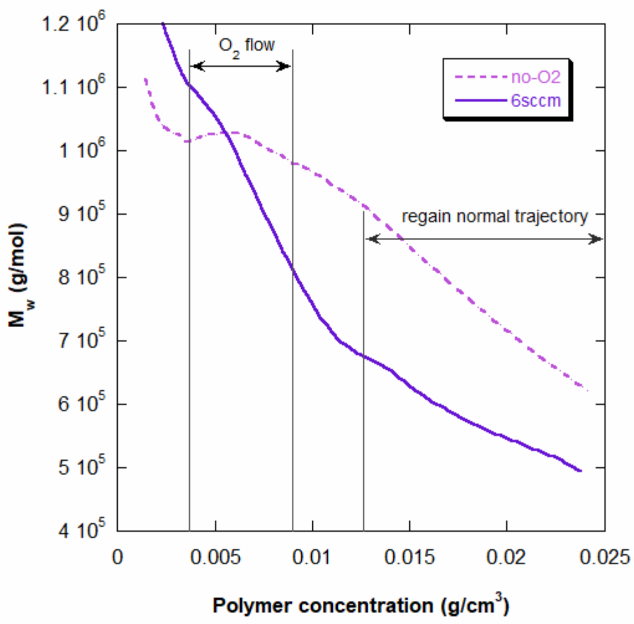

Figure S5. Cumulative weight average molecular weight (M_w) for polymerization of Am with 6 sccm airflow and with no O_2 (reactions 12A and 13A in Table 1, respectively).

S3. Full estimation of Am catalytic efficiency for case (i)

The time-dependent signatures of $[O_2]$ in the reactor allow for some modeling of the process, provide further evidence of the reactions in the above kinetic model, and an estimate of the total amount of O_2 consumed during the air flow, and after it is stopped. The following derivation is based on these two assumptions: 1) the reactor is vented to atmosphere via a needle, so it is assumed that the headspace of the reactor remains at atmospheric pressure throughout. Because the needle is small-bore this assumption may not be strictly true, and there could be some small, transient pressure increases in the headspace as air flows into the reactor. 2) the volume of liquid in the reactor over the conversion plateau does not change significantly. It was found experimentally that the mixing of inflowing O_2 into the headspace and dissolution into the reactor water, initially N_2 purged, does not occur at the same rate (see Figure SI4 above).

Consider the case of instantaneous, ideal mixing of O_2 flowing with a molar flow rate Q (mol/s) into a vented vessel of volume V originally filled with N_2 at atmospheric pressure. Then

$$\frac{d[O_2]}{dt} = \alpha([O_2]_{sat} - [O_2]) \tag{SI1}$$

for which Equation 1 is the solution, and α is the rate constant given by Equation 2, for ideal mixing. $[O_2]_{sat}$ is the concentration of O_2 in air at a given temperature. In the following $[O_{2,H}]$ and $[O_{2,R}]$ represent the concentrations of O_2 in the headspace and dissolved in the aqueous reactor liquid, respectively.

All measurements of $[O_2]$ both in the headspace and reactor water followed Equation 1 nearly perfectly, but with values of α in the reactor not dependent only on Q and V, as in Equation 2. In particular, dissolution of the O_2 into water yields α_R about 6.5x lower than α_H in the headspace, at a 500ml/100ml solution/headspace volume ratio. This difference in rates means that $[O_2]$ in the headspace will more quickly get closer to its saturation value than $[O_2]$ in the liquid, allowing the headspace O_2 to act as a source term of O_2 flowing into the liquid, as long as $[O_{2,R}]<[O_{2,R}]_{sat}$, where $[O_{2,R}]_{sat}$ is the saturation for dissolved O_2 in the reactor liquid. This is clearly seen in Figure 3, where the backflow from headspace to liquid was measured directly after airflow was stopped, when no reaction was occurring. In Figure 6 the effect is dramatic during the RAFT reaction, where the $[O_{2,R}]$ increased strongly, well after the airflow stopped due to backflow from headspace to liquid and the suppression of the catalytic O_2 elimination cycle observed in RAFT. Even in the RP case (i), such as in Figure 2, there was usually a short period of a small O_2 increase after the airflow stopped, due to the headspace backflow, but this was quickly overwhelmed in the RPs by the O_2 elimination cycle, causing $[O_{2,R}]$ to decrease after reaching a maximum. Because $[O_{2,H}]_{sat} = 58[O_{2,R}]_{sat}$, at 50 °C, even a relatively small headspace (V_H~100cm^3 versus V_R = 500cm^3) holds ample O_2 for backflow into the reactor.

The effect of the headspace backflow of O_2 into the reactor can be expressed with an experimental rate constant β, which is related to the diffusivity of O_2 from the headspace and dissolution into the reactor liquid, and the amount of interfacial area separating the liquid from the headspace. This leads to a gain of O_2 in the reaction given by

$$\frac{d[O_{2,R}]}{dt}_{headspace\ backflow} = -\frac{d[O_{2,H}]}{dt}_{headspace\ backflow} = \beta\left(\epsilon[O_{2,H}] - [O_{2,R}]\right) \tag{SI2}$$

where $\epsilon = \frac{[O2,R]sat}{[O2,H]sat} = 0.017$ is taken to scale the headspace $[O_{2,H}]$ to levels of $[O_{2,R}]$. The difference drives headspace O_2 into the reactor liquid. At equilibrium there is no further headspace backflow of O_2 into the reactor liquid.

The kinetic model predicts that when the Am RP reaction occurs, O_2 flowing into the reactor is swept into the process, both in the O_2 elimination catalyzed by Am* in case (i), and as an inhibitory chain termination agent below $[O_2]_t$ in cases (i) and (ii). Hence, in case (i), the rate of increase of $[O_2]$ (α) in the reactor liquid during a polymerization reaction should be less than when O_2 flows into a non-reacting aqueous mixture, since $[O_2]$ is chemically eliminated during the reaction, but not during a flow into non-reacting aqueous phase.

This prediction for case (i) is born out clearly in Figure 3, where the initial build-up rate of O_2 in the reaction, $[O_{2,R}]$, is about 13x slower than when O_2 flows at the same rate into non-reacting aqueous reactor content; i.e. the effect of O_2 elimination during the reaction is very strong. From Equation 9, O_2 in the reactor is chemically eliminated at a rate given by

$$\frac{d[O_{2,R}]}{dt}_{O2\ elimination\ reaction} = -[O_{2,R}]\left(k_{12}[A*] + k_{t,2R}[R*]\right) \qquad \text{(SI3)}$$

Combining the headspace backflow, $q(t) = \alpha_R\left([O_{2,R}]_{sat} - [O_{2,R}]\right)$, and O_2 elimination terms while the airflow is on (α_R>0), gives the following for reactor O_2

$$\frac{d[O_{2,R}]}{dt} = \alpha_R\left([O_{2,R}]_{sat} - [O_{2,R}]\right) + \beta\left(\epsilon[O_{2,H}] - [O_{2,R}]\right) - [O_{2,R}]\left(k_{12}[A*] + k_{t,2R}[R*]\right) \qquad \text{(SI4)}$$

When the airflow is cut off ($\alpha_R = 0$) then

$$\frac{d[O_{2,R}]}{dt} = \beta\left(\epsilon[O_{2,H}] - [O_{2,R}]\right) - [O_{2,R}]\left(k_{12}[A*] + k_{t,2R}[R*]\right) \qquad \text{(SI5)}$$

For O_2 in the headspace while the reaction is occurring

$$\frac{d[O_{2,H}]}{dt} = \alpha_H'\left([O_{2,H}]_{sat} - [O_{2,H}]\right) - \beta\left(\epsilon[O_{2,H}] - [O_{2,R}]\right) \qquad \text{(SI6)}$$

where α_H' is the rate when the reaction occurs, and $\alpha_H' < \alpha_H$, since dissolved O_2 is being eliminated in the reaction liquid and reduces the flow rate of O_2 to the headspace.

The two equations are coupled and so are most easily solved numerically. For a quick analytical estimate, however, it can be approximated that, because $[O_{2,H}]$ in the headspace increases much more rapidly than $[O_{2,R}]$, $\epsilon[O_{2,H}] \sim \epsilon[O_{2,H}]_{sat} = [O_{2,R}]_{sat}$, and that the radical concentrations [Am*] and [R*] are approximately constant, c,

$$c = \left(k_{12}[Am*] + k_{t,2R}[R*]\right) \qquad \text{(SI7)}$$

then Equation SI3 is solvable and gives the first order solution for the airflow period $[O_{2,R}]_I(t)$, where t_f is the time the air flow stopped

$$0 \le t \le t_f, \ \ [O_{2,R}]_I(t) = \frac{(\alpha_{H'}+\beta)}{\alpha_{H'}+\beta+c}[O_{2,R}]_{sat}\left(1-e^{-(\alpha_{H'}+\beta+c)t}\right) \tag{SI8}$$

This implies that if the air flow is kept on long enough $[O_{2,R}]_I(t)$ reaches a plateau lower than $[O_{2,R}]_{sat}$, given by

$$\lim_{t\to\infty}[O_{2,R}]_I(t) \ = \frac{(\alpha_{H'}+\beta)}{\alpha_{H'}+\beta+c}[O_{2,R}]_{sat} \tag{SI9}$$

When the airflow is cut off at t_f the new solution, $[O_{2,R}]_{II}(t)$ is again first order, and must match the first solution at t = t_f, that is

$$[O_{2,R}]_I(t_f) = [O_{2,R}]_{II}(t_f) \tag{SI10}$$

So that

$$t \ge t_f, [O_{2,R}]_{II}(t_f) = [O_{2,R}]_{sat}\left\{\frac{\beta}{\beta+c}\left(1-e^{-(\beta+c)(t-t_f)}\right)+\frac{(\alpha_{H'}+\beta)}{\alpha_{H'}+\beta+c}[O_{2,R}]_{sat}\left(1-e^{-(\alpha_{H'}+\beta+c)t_f}\right)e^{-(\beta+c)(t-t_f)}\right\} \tag{SI11}$$

This shows that O_2 is not entirely eliminated from the reaction liquid, and ends at a value

$$\lim_{t\to\infty}[O_{2,R}]_{II}(t) \ = \frac{\beta}{\beta+c}[O_{2,R}]_{sat} \tag{SI12}$$

Figure 3 shows that $\lim_{t\to\infty}[O_{2,R}]_{II}(t)$ is near the lower limit of sensitivity of the O_2 probe, so that c>>β, for the reaction conditions determining c and reactor geometry partially determining β.

The total amount of O_2 eliminated chemically can be estimated for a fixed air flow rate by subtracting from the total amount of O_2 at saturation with no reaction the amount of $[O_{2,R}]$ measured at an equivalent time during an RP. This difference is approximately the amount of O_2 chemically eliminated over the period. Once the air flow stops, the remaining dissolved O_2 will be driven down to the O_2 reaction turn-off threshold, $[O_{2,R}]_t$ by the reaction, and even lower. Since $[O_{2,R}]_t<<[O_{2,R}]_{sat}$, the amount of O_2 consumed is approximately equal to $[O_{2,R}]_{sat}$. If the airflow continues until the reduced O_2 plateau height of Equation SI8 $\frac{(\alpha_{H'}+\beta)}{\alpha_{H'}+\beta+c}[O_{2,R}]_{sat}$, then the reaction will continue to consume O_2 at a steady rate. Taking β<<c, α_H' and, yields

$$\frac{\alpha_{H'}}{\alpha_{H'}+c} = \frac{[O_{2,R}]_{plateau}}{[O_{2,R}]_{sat}} \tag{SI13}$$

Reaction 11A in Table 2 used a flow rate of 1,000 sccm for a period of 16,000 s, reaching a $[O_{2,R}]$ plateau of 5.68×10^{-6} g/cm^3. Without a reaction, saturation is 6.56×10^{-6} g/cm^3. This gives $\frac{\alpha_{H'}}{\alpha_{H'}+c} = \frac{[O_{2,R}]_{plateau}}{[O_{2,R}]_{sat}} = 0.87$. Fitting the initial rise time below, gives $\alpha_H' + c = 0.0031$, so that α_H' = 0.0027 1/s, and c = 4×10^{-4} 1/s. The amount of O_2 consumed by the elimination reaction on the plateau is

$$\Delta[O_{2,R}] \sim c[O_{2,R}]\Delta t \sim 1.14x10^{-6}\frac{mol}{cm^3}$$

using $\Delta t = 16{,}000\ s$ and $[O_{2,R}]$ = 5.68x10^{-6} g/cm^3/32 g/mol = 1.78x10^{-7} mol/cm^3. If one Am were consumed per elimination of a single O_2 then the change in [Am] over the 16,000 s is ~8x10^{-5} g/cm^3. With a baseline noise of ~+/- 3.6x10^{-5} g/cm^3, such change would be measurable, but is not found.

Another way of quickly estimating the catalytic efficiency in case (i) is to consider the number of free radicals generated over 16,000 s from KPS decay, at 50 ^oC, which equals to only 8.7x10^{-8} mol/cm^3 of KPS radicals. Since the amount of O_2 eliminated over 16,000 s is ~ 1.14x10^{-6} mol/cm^3, a single I* must suffice to eliminate at least 13 molecules of O_2. As proved by the control experiments (see Figures SI3 and 3), this elimination is mediated by Am at this low initiator concentration, so each Am must be able to go through at least 13 catalytic cycles, and likely more, before being used up in a side reaction (e.g. termination).

The prediction from the approximation obtained by eliminating [$O_{2,H}$] from the balance equation for [$O_{2,R}$](t) is that it will have a first order growth while the air flow is on, and will subsequently decay to a small but finite value when the airflow stops. This is qualitatively the trends seen for [$O_{2,R}$](t) during RP. If this approximation is dropped and the coupled equations integrated numerically (not shown), including the changing reactor liquid volume due to ACOMP extraction, the rounding of the decay profile with an inflection point, appears.

As regards shape, at first sight the O_2 behavior in Figure 3 appears fairly symmetric. Upon closer inspection and examining many such O_2 profiles suggests that the buildup to the maximum is first order, as in pure water, albeit at a much lower rate, and that the downside is initially Gaussian, before becoming exponential. While there were problems with mixing and homogeneity of O_2 as far as obtaining accurate O_2 data is concerned, the data from numerous experiments allow fair estimates of O_2 threshold values, and other features, such as shape, rise time, and peak values.
